# A Macrophage Subversion Factor Is Shared by Intracellular and Extracellular Pathogens

**DOI:** 10.1371/journal.ppat.1004969

**Published:** 2015-06-16

**Authors:** Claudine Belon, Chantal Soscia, Audrey Bernut, Aurélie Laubier, Sophie Bleves, Anne-Béatrice Blanc-Potard

**Affiliations:** 1 Laboratoire de Dynamique des Interactions Membranaires Normales et Pathologiques, Université de Montpellier, CNRS-UMR5235, Montpellier, France; 2 CNRS & Aix-Marseille Université, Laboratoire d’Ingénierie des Systèmes Macromoléculaires (UMR7255), Marseille, France; Northwestern University, UNITED STATES

## Abstract

Pathogenic bacteria have developed strategies to adapt to host environment and resist host immune response. Several intracellular bacterial pathogens, including *Salmonella enterica* and *Mycobacterium tuberculosis*, share the horizontally-acquired MgtC virulence factor that is important for multiplication inside macrophages. MgtC is also found in pathogenic *Pseudomonas* species. Here we investigate for the first time the role of MgtC in the virulence of an extracellular pathogen, *Pseudomonas aeruginosa*. A *P*. *aeruginosa mgtC* mutant is attenuated in the systemic infection model of zebrafish embryos, and strikingly, the attenuated phenotype is dependent on the presence of macrophages. In *ex vivo* experiments, the *P*. *aeruginosa mgtC* mutant is more sensitive to macrophage killing than the wild-type strain. However, wild-type and mutant strains behave similarly toward macrophage killing when macrophages are treated with an inhibitor of the vacuolar proton ATPase. Importantly, *P*. *aeruginosa mgtC* gene expression is strongly induced within macrophages and phagosome acidification contributes to an optimal expression of the gene. Thus, our results support the implication of a macrophage intracellular stage during *P*. *aeruginosa* acute infection and suggest that *Pseudomonas* MgtC requires phagosome acidification to play its intracellular role. Moreover, we demonstrate that *P*. *aeruginosa* MgtC is required for optimal growth in Mg^2+^ deprived medium, a property shared by MgtC factors from intracellular pathogens and, under Mg^2+^ limitation, *P*. *aeruginosa* MgtC prevents biofilm formation. We propose that MgtC shares a similar function in intracellular and extracellular pathogens, which contributes to macrophage resistance and fine-tune adaptation to host immune response in relation to the different bacterial lifestyles. In addition, the phenotypes observed with the *mgtC* mutant in infection models can be mimicked in wild-type *P*. *aeruginosa* strain by producing a MgtC antagonistic peptide, thus highlighting MgtC as a promising new target for anti-virulence strategies.

## Author Summary

Pathogenic bacteria have to resist host immune response and MgtC is used by several intracellular pathogens to promote bacterial multiplication inside macrophages. Here we investigated MgtC’s role in the virulence of an extracellular pathogen, *Pseudomonas aeruginosa*. A *P*. *aeruginosa mgtC* mutant is attenuated in zebrafish embryos, but only in the presence of macrophages. Moreover, this mutant is more rapidly killed by macrophages than the wild-type strain. Both phenotypes can be mimicked upon production of a MgtC antagonistic peptide in wild-type *Pseudomonas* strain. MgtC thus provides a singular example of a virulence determinant that promotes strategies to subvert the antimicrobial behavior of macrophages, in both intracellular and extracellular pathogens and our results support an intramacrophage stage during in *P*. *aeruginosa* acute infection, as well as an interplay between MgtC role and phagosome acidification. In addition, *P*. *aeruginosa* MgtC is required for growth in Mg^2+^ deprived medium, a property shared by MgtC factors from intracellular pathogens, and limits biofilm formation. MgtC may share a similar function in intracellular and extracellular pathogens, with an outcome adapted to the different bacterial lifestyles

## Introduction

Pathogenic bacteria have developed numerous strategies to adapt to host environment and common strategies can be used by pathogens that share similar lifestyle or similar environmental niches. MgtC is a virulence factor common to several intracellular pathogens [1]. It was first described in *Salmonella enterica* serovar Typhimurium (*S*. Typhimurium) as required for intramacrophage multiplication and systemic infection in mice [2–4]. MgtC appears as a singular factor that promotes pathogenicity by inhibiting the *Salmonella*'s own F_1_F_o_ ATP synthase [5]. MgtC has been shown to directly interact with F_1_F_o_ ATP synthase, thereby altering its ability to translocate protons and to couple translocation to ATP synthesis. *Salmonella mgtC* is highly regulated both at the transcriptional and post-transcriptional levels. This regulation includes a positive regulation by Mg^2+^ deprivation [6] and by an increase in cytosolic ATP [7] as well as a negative regulation by the MgtR peptide [8]. MgtC was also described as a critical factor for the intramacrophage growth of *Mycobacterium tuberculosis*, *Brucella suis*, *Yersinia pestis*, *Burkholderia cenocepacia* and *Salmonella enterica* serovar Typhi, being a virulence factor in a mouse model for *M*. *tuberculosis* and *B*. *cenocepacia* [9–13]. More recently, a contribution for MgtC in phagocytosis has been uncovered in *Mycobacterium marinum* [14]. In addition, MgtC has been involved in adaptation to low Mg^2+^ environments in these many pathogens [1]. However, the role of MgtC in low Mg^2+^ environments can be dissociated from its role in macrophages, suggesting that MgtC has a dual function [15].

Genes encoding MgtC-like proteins are found in a limited number of eubacterial genomes and phylogenetic analysis suggested that *mgtC* has been acquired by horizontal gene transfer repeatedly throughout bacterial evolution [16]. Alignment of MgtC-like proteins as well as hydrophobicity pattern clearly define two domains: an hydrophobic N-terminal part, highly conserved in all MgtC-like proteins, and a soluble C-terminal part that is much more variable, but specifically conserved in the subgroup of MgtC proteins from intracellular pathogens [16]. Noticeably, this phylogenetic subgroup also contains two MgtC-like proteins, PA4635 and PA2558, from *Pseudomonas aeruginosa* [16], suggesting a potential common function with proteins from intracellular pathogens. Because of this phylogenetic clustering, we have previously investigated whether the *P*. *aeruginosa mgtC*-like genes can complement a *S*. Typhimurium Δ*mgtC* strain [15]. *PA4635* fully complemented the *Salmonella mgtC* mutant for growth in low Mg^2+^ medium but not in macrophages. On the other hand, *PA2558* failed to complement the *Salmonella* Δ*mgtC* mutant for growth both in low Mg^2+^ medium and in macrophages. In addition, amino-acid sequence alignment of MgtC-like proteins from *Salmonella* phylogenetic subgroup indicated that some conserved residues important for *Salmonella* MgtC function are not conserved in PA2558 [15]. Taken together, these results suggested that only PA4635 shares functional properties with the *Salmonella* MgtC and is herein referred as *P*. *aeruginosa* MgtC.

In the present study, we investigated for the first time the role of MgtC in an extracellular pathogen, *P*. *aeruginosa*. The environmental bacterium and opportunistic human pathogen *P*. *aeruginosa* is a major cause of mortality in cystic fibrosis (CF) patients. Interestingly, MgtC has been highlighted as a horizontally-acquired gene shared by several opportunistic bacteria infecting CF patients [17]. *P*. *aeruginosa* virulence and resistance to treatment is largely due to its ability to form biofilms, whose stability can be affected by extracellular cations [18,19]. *P*. *aeruginosa* is known to impair host phagocytic functions in chronic lung infections of CF patients. However, macrophages can play a protective role against *P*. *aeruginosa* infection in a systemic model of infection in zebrafish embryos, where *P*. *aeruginosa* has been shown to be phagocytosed by macrophages [20]. In addition, despite the fact that *P*. *aeruginosa* is an extracellular pathogen, an intracellular step in airway epithelial cells might occur before the formation of biofilm during the acute phase of infection [21,22]. In the present study, we have constructed a *P*. *aeruginosa mgtC* mutant and have analysed its infection phenotype in animal and cellular models, as well as biofilm formation. We also investigated *mgtC* gene expression *in vitro* and *in cellulo*. In addition, MgtR, a peptide that has been proposed as a MgtC antagonist in *Salmonella* [8] has been heterologously produced in a wild-type *P*. *aeruginosa* strain (*P*. *aeruginosa* does not encode a MgtR homologue). We establish that MgtC contributes to *Pseudomonas* acute infection and resistance to macrophage killing, thus being a factor that subverts the antimicrobial behavior of macrophages both in intracellular and extracellular pathogens.

## Results

### Phylogenetic analysis of MgtC-like proteins from *Pseudomonas* species

**Fig 1:**
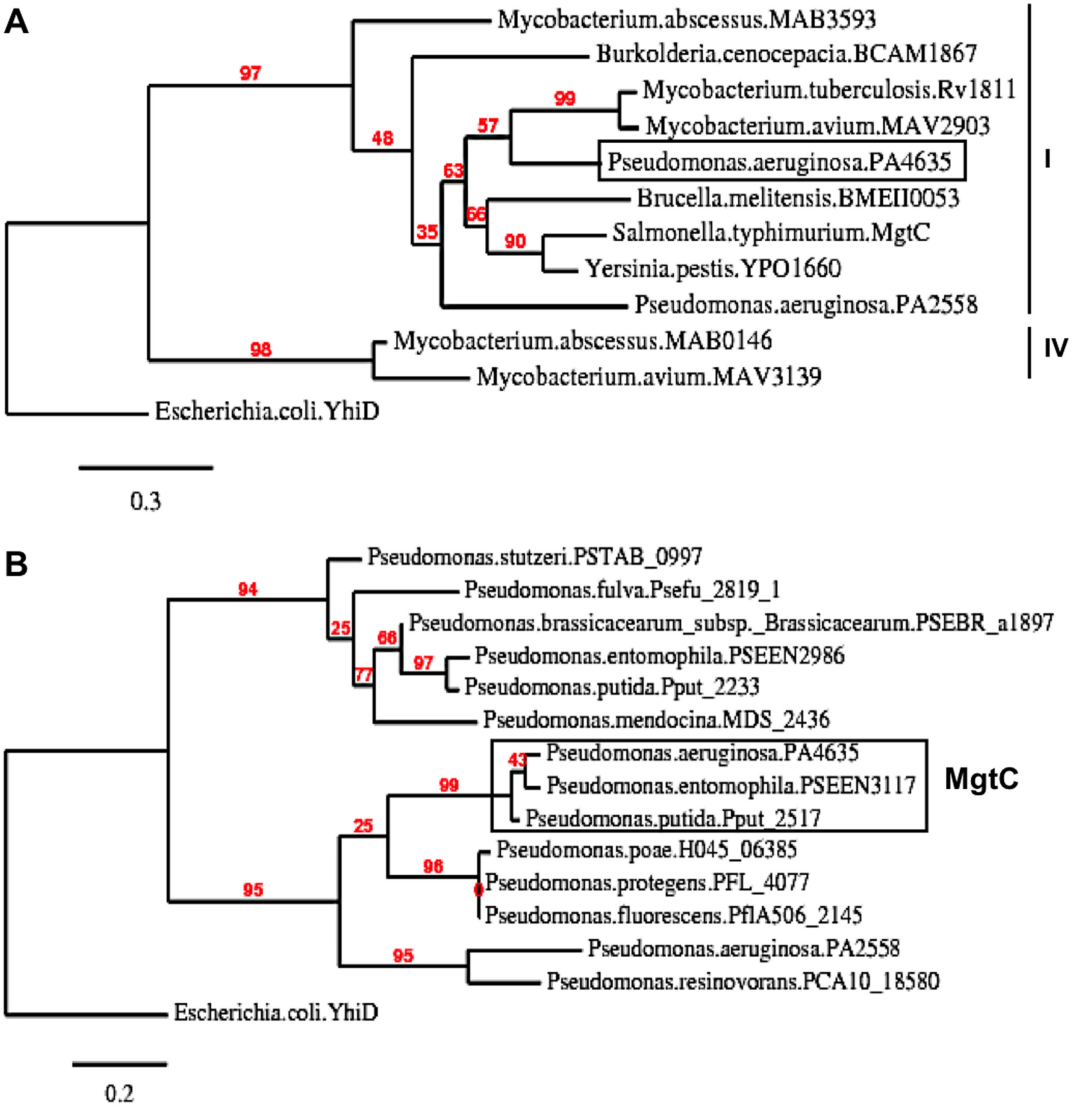
Phylogenetic analysis of *Pseudomonas* MgtC-like proteins. (A) Phylogenetic tree of MgtC-like proteins from bacterial pathogens that survive in macrophages for which MgtC role in virulence has been studied (*S*. Typhimurium, *M*. *tuberculosis*, *B*. *suis*, *B*. *cenocepacia*, *Y*. *pestis*) and opportunist bacterial pathogens found in CF patients (*P*. *aeruginosa*, *B*. *cenocepacia*, *M*. *avium*, *M*. *abscessus*). Several bacteria, including *P*. *aeruginosa*, encode two MgtC-like proteins. Phylogenetic analyses were carried out with the “Phylogeny.fr” web server (http://www.phylogeny.fr) [57] using MUSCLE, PhyML and TreeDyn softwares. Numeration of phylogenetic groups follows a previous analysis [58]. *Escherichia coli* YhiD is used as a distantly related MgtC-like protein [16] to root the tree. (B) Phylogenetic tree of MgtC-like sequences recovered from *Pseudomonas* genomes. MgtC-like protein sequences were recovered from BlastP analysis using PA4635 sequence on completed genomes from the *Pseudomonas* genome database (http://v2.pseudomonas.com/) [23]. PA4635 is conserved (>99% identity) in the 10 *P*. *aeruginosa* genomes other than PAO1 found in the *Pseudomonas* database, including the divergent strain PA7. Besides *P*. *aeruginosa* genomes, MgtC is found in *P*. *entomophila* and *P*. *putida* (>85% identity with PA4635). Other genomes harbors MgtC-like proteins that differ from PA4635 (<40% identity with PA4635).

The genome of *P*. *aeruginosa* strain PAO1 encodes two MgtC-like proteins, PA4635 and PA2558, which both belong to the same phylogenetic subgroup as *Salmonella* MgtC [16]. In Fig 1A, we provided a phylogenetic analysis of this subgroup that focused on proteins from bacterial pathogens able to replicate in macrophages for which MgtC role in virulence has been studied, as well as proteins from opportunistic bacteria infecting CF patients.

As *mgtC* sequences may have been acquired by horizontal gene transfer [16], we investigated the distribution of *mgtC* coding sequence in *Pseudomonas* genomes and performed phylogenetic analysis. MgtC appeared to be conserved in all *P*. *aeruginosa* strains present in the *Pseudomonas* genome database [23,24], including the atypical PA7 strain [24]. In addition, MgtC was found in the insect pathogen *Pseudomonas entomophila* as well as the closely related *Pseudomonas putida* (> 85% identity with PA4635, Fig 1B). In contrast, other *Pseudomonas* species harbor MgtC-like proteins that largely differ from PA4635 (below 40% identity, Fig 1B) or do not harbor any MgtC-like protein (*Pseudomonas syringae*). This analysis indicates a strong association of MgtC with *Pseudomonas* species that are pathogenic for humans and insects, suggesting a putative role during animal/human host infection.

To address the role of MgtC in *P*. *aeruginosa*, we constructed an in-frame deletion in *mgtC* (*PA4635*) to avoid any polar effect on the expression of the downstream *PA4636* gene (S1 Fig). The resulting Δ*mgtC* mutation was complemented by a single copy of the *mgtC* gene with its own promoter inserted into the unique *attB* site in strain PAO1 (S1 text).

### MgtC is important for *P*. *aeruginosa* virulence in the *Danio rerio* infection model in a macrophage-dependent manner

**Fig 2:**
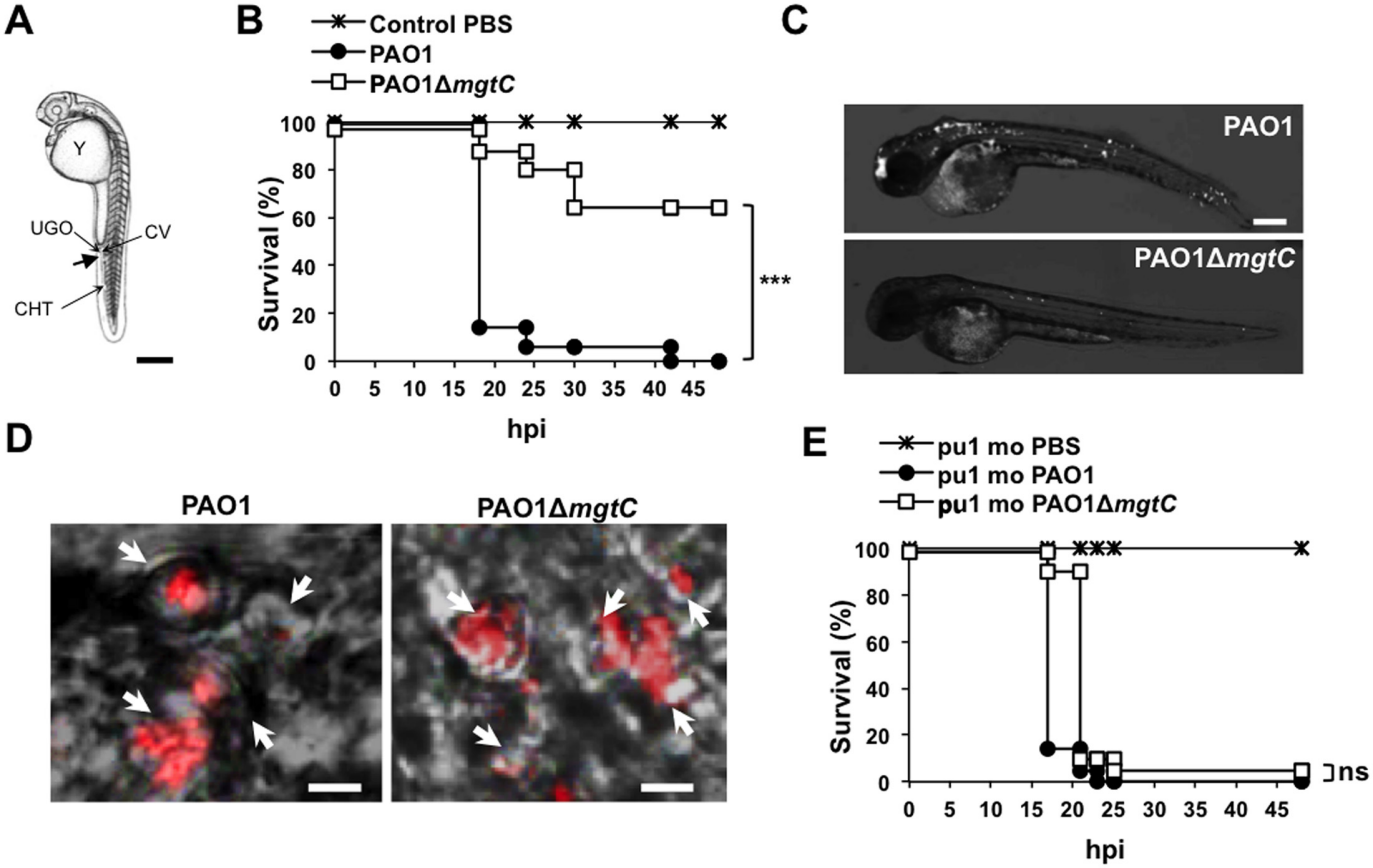
Infection of zebrafish embryos with the *P*. *aeruginosa mgtC* mutant. (A) Diagram of 30 hours post-fertilization (hpf) zebrafish embryo showing the injection site used in this study (arrow). All injections were done in the caudal vein (CV) just behind to the urogenital opening (UGO). Y: Yolk, CHT: Caudal Hematopoietic Tissue. Scale bar, 100 μm. (B) Survival curves of embryos infected with PAO1 wild-type stain or PAO1 Δ*mgtC* mutant and PBS-injected (control). Approximately 1200–1400 CFU *P*. *aeruginosa* were microinjected into the caudal vein (n = 20 per group). Results are expressed as the percentage of surviving on each hour post-infection. Representative results of three biologically independent replicates are shown. Embryos are significantly more susceptible to infection with PAO1 wild-type than Δ*mgtC* mutant (*P*<0.001). (C) 30 hpf embryons are intravenously infected with PAO1 or Δ*mgtC* mutant expressing mCherry. Representative fluorescence microscopy images of 18 hpi embryos infected with PAO1 (top panel) or PAO1 Δ*mgtC* mutant (bottow panel) are shown (1200–1400 CFU). Scale bar, 200 μm. (D) 30 hpf zebrafish embryos are intravenously infected with mCherry-expressing PAO1 (left panel) or PAO1 Δ*mgtC* mutant (right panel) and imaged by confocal microscopy. Arrows indicate maximum intensity projection of macrophages that phagocytose bacteria close to the site of injection at 1 hpi. Scale bar, 10 μm. (E) Survival curves of *pu*.*1* morphant (*pu*.*1* mo) embryos (n = 20 each) infected with 900 CFU of PAO1 wild type stain or PAO1 Δ*mgtC* mutant compared to the PBS- injected control. Representative results of three biologically independent replicates are shown. No statistically significant difference is obtained between wild type-infected embryos and Δ*mgtC* mutant-infected embryos (ns: non significant).

To evaluate the role of MgtC in *P*. *aeruginosa* virulence, we used the zebrafish (*Danio rerio)* embryo model. This model, which has been used for various intracellular and extracellular bacterial pathogens, is a model of choice to investigate the contribution of cells from the innate immune system during infection [25]. The *Danio* has been successfully used to monitor the role of *P*. *aeruginosa* determinants in virulence based on survival curves of larvae infected with mutants deficient in type III secretion system (T3SS) or Quorum Sensing [20,26]. Bacteria producing a fluorescent protein were injected intravenously in the caudal vein of embryos (Fig 2A) at 30 hours post-fertilization, a time where macrophages, but not neutrophils, are fully functional and capable of engulfing invading bacteria [27]. The survival curves of infected embryos indicated that MgtC is a critical virulence determinant in this model since the *mgtC* mutant is significantly attenuated as compared to the wild-type PAO1 strain (Fig 2B). In agreement, fluorescence microscopy of 18 hpi infected embryos showed a lower bacterial burden with PAO1 strain than *mgtC* mutant (Fig 2C).

Confocal microscopy at early time after injection allowed us to visualize bacteria phagocytosed by macrophages close to the site of injection (Fig 2D). Macrophages can be depleted from zebrafish embryos with a validated method that uses antisens oligonucleotides (morpholinos) against the myeloid transcription factor gene *pu*.*1* [28,29]. Macrophage-depleted zebrafish embryos have been shown to be hypersusceptible to *P*. *aeruginosa* infection [20]. In addition, it has been shown that macrophage depletion restored the virulence of the attenuated T3SS mutant in zebrafish embryos [20]. We carried out experiments with Tg(*mpeg1*::*mCherry*) embryos in which macrophages are visualized as red cells [29]. Therefore, macrophage depletion can be checked upon *pu*.*1* morpholinos injection (S2 Fig). Interestingly, the survival curve of macrophage-depleted embryos was similar upon infection with wild-type and mutant strains (Fig 2E and S3 Fig), indicating that macrophage depletion suppresses the difference between wild-type and mutant strain. Taken together, these results suggest that MgtC acts by protecting *P*. *aeruginosa* against macrophages.

### MgtC contributes to *P*. *aeruginosa* resistance to macrophage killing in J774 macrophages

**Fig 3:**
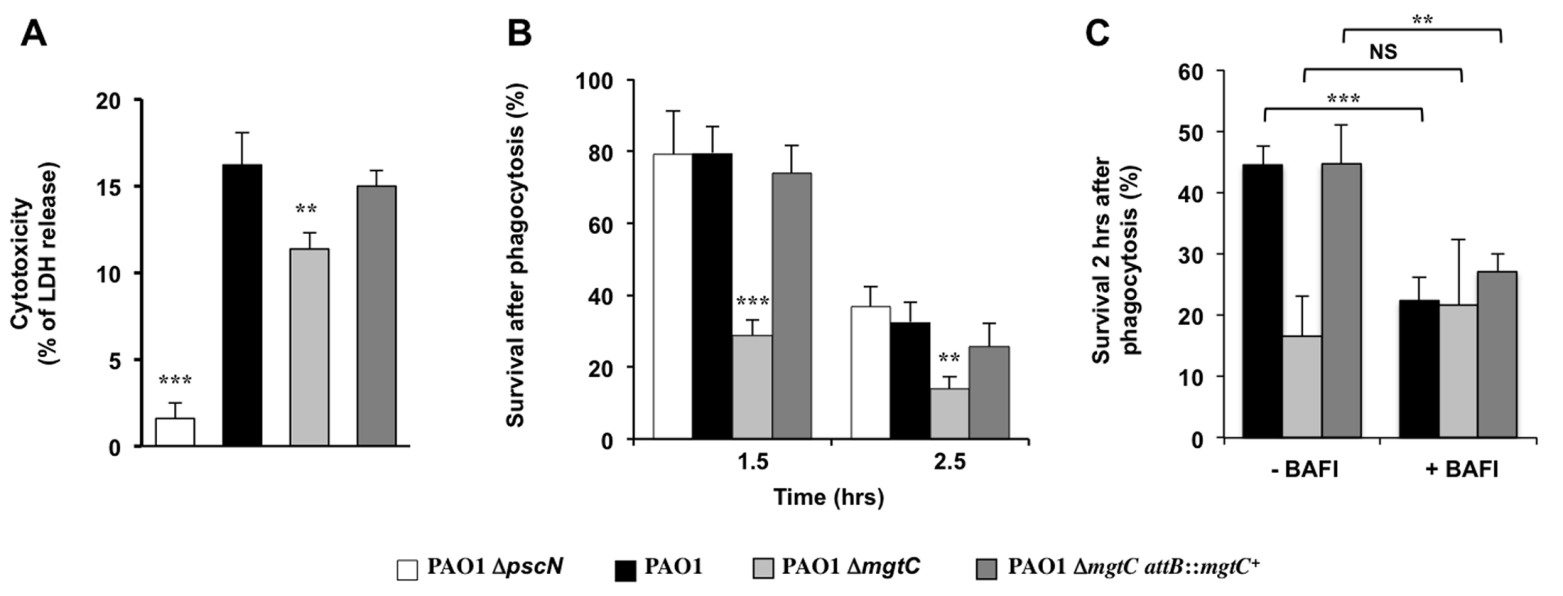
Behaviour of *mgtC* mutant towards J774 macrophages. (A) Strain cytotoxicity was evaluated using a LDH assay. A T3SS mutant (Δ*pscN*) is included as negative control. Error bars correspond to standard deviations from three independent experiments. (B) Survival of bacteria upon phagocytosis. Results are expressed as percentage of surviving bacteria at the indicated time comparatively to the number of bacteria internalized after 30 min of phagocytosis. Error bars correspond to standard errors (SE) from five independent experiments. (C) Effect of inhibition of vacuolar proton ATPase on bacterial survival. The survival of bacteria was measured from macrophages treated or not with bafilomycin A1. Error bars correspond to standard errors (SE) from three independent experiments. In all panels the asterisks indicate *P* values (Student’s t test, **P <0.01, ***P <0.001).

The virulence of *P*. *aeruginosa* is associated with its ability to resist the innate immune system notably because of a cytotoxic action towards macrophages and the T3SS plays a major role in *Pseudomonas* cytotoxicity [30]. We thus tested the cytotoxicity of the *mgtC* mutant on J774 macrophages and showed that the mutant is slightly but significantly less cytotoxic than the wild-type strain or the complemented strain (Fig 3A). However, the reduction of cytotoxicity was much lower than the one observed with a T3SS mutant (Fig 3A). Given our results in zebrafish embryos, we hypothesized that MgtC may play a role in the ability of *P*. *aeruginosa* strains to resist killing by phagocytic cells. We therefore tested the sensitivity of *P*. *aeruginosa* strains to the bactericidic activity of J774 macrophages. Whereas the T3SS mutant behaves similarly than the wild-type strain towards the killing by macrophages after phagocytosis, our results indicated that the *mgtC* mutant is more sensitive to macrophage killing than the wild-type or complemented strain, with a striking difference at time 1.5 hr (Fig 3B). The survival decrease of the wild-type strain at the second time point may account for a slower killing, but may also be due in part to the damage of infected macrophages.

*Salmonella* MgtC can inhibit the bacterial F-ATP synthase and modulate physiological ATP levels and cytosolic pH, which has been proposed to play a role in the ability of *Salmonella* strain to replicate in macrophages [5]. Moreover, acidification of the phagosome has been proposed to promote *Salmonella* MgtC production by increasing bacterial ATP level [7]. The acidification of the phagosome is dependent on the activity of the host vacuolar ATPase, which can be specifically inhibited by the inhibitor bafilomycin A1 [31]. To decipher the contribution of phagosome acidification in the intramacrophage role of *Pseudomonas* MgtC protein, macrophage infection was performed in presence of bafilomycin A1 (Fig 3C). The addition of bafilomycin A1 did not affect significantly the mutant strain whereas the survival rate of the PAO1 strain or the complemented strain was significantly reduced. As a consequence, the survival rate of wild-type and mutant strains did not differ when cells are treated with vacuolar ATPase inhibitor.

**Fig 4:**
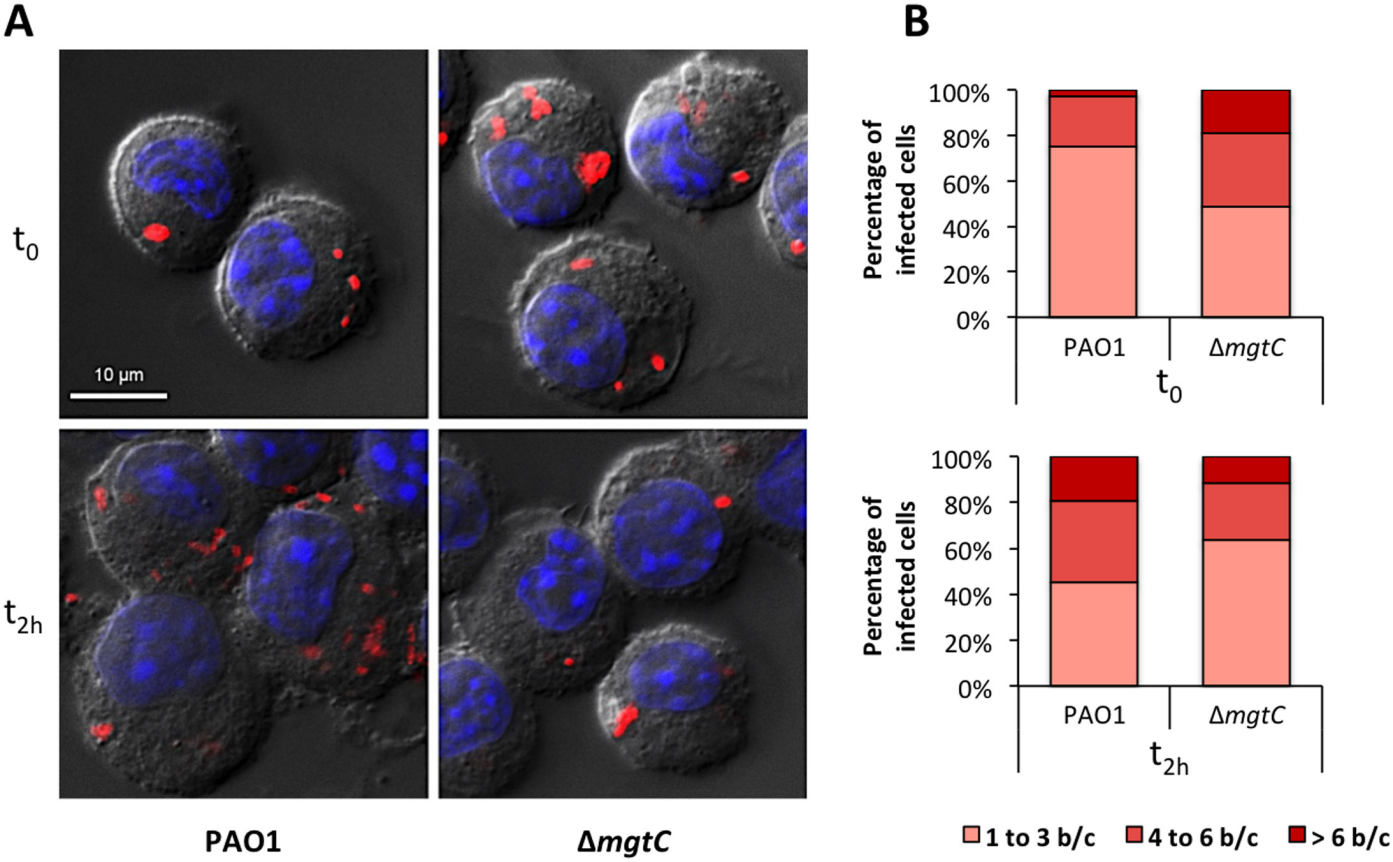
Visualisation and quantification of intracellular bacteria in fixed macrophages. Macrophages infected with PAO1 or Δ*mgtC* mutant expressing mCherry were fixed after phagocytosis and 20 min (t_0_) or 2 hours (t_2h_) treatment with amikacin. (A) Visualization of intracellular bacteria. The images shown were obtained by making a maximum projection of 12 central planes from a Z-stack. The merged image shows Differential Interference Contrast (DIC), nucleus staining (blue) and bacteria expressing mCherry (red). (B) Count of the number of bacteria in infected macrophages from images obtained with the maximum projection. The numbers of bacteria per cell (b/c) were classified in three groups and percentage of each class is shown. Count is done from at least 20 cells and results are expressed as means from three independent experiments.

To visualize intracellular bacteria upon infection of J774 cells, we imaged slides of fixed macrophages infected with fluorescent bacteria (Fig 4A). Moreover, bacterial counts on images were made at time 0 and 2 hrs after phagocytosis (Fig 4B). Our results indicated that the number of bacteria per cell after phagocytosis is slightly higher for the mutant strain than for the wild-type strain. We further quantified the phagocytosis rate with CFUs counts and showed that the *mgtC* mutant is phagocytosed at a slightly but significantly higher rate than the wild-type strain (S4 Fig). The evolution of the patterns between time 0 and time 2 hrs (Fig 4B) indicated that the bacterial number per cell rather increased with the wild-type but not with the mutant. These experiments, which do not discriminate between live and dead/dying bacteria, suggested a survival of wild-type bacteria with limited bacterial multiplication.

We also investigated the behavior of the *mgtC* mutant towards non-phagocytic cells that can be targeted by the pathogen during the infection [22]. Gentamycin protection assays performed to measure internalisation in HeLa cells did not show significant difference between wild-type and *mgtC* mutant strains (S4 Fig).

Cumulatively, *ex vivo* experiments indicate that, similarly to intracellular pathogens, the MgtC virulence determinant of *P*. *aeruginosa* plays a role towards macrophages. Wild-type bacteria resisted better to phagocytosis and to bacterial killing by the phagosome than the *mgtC* mutant. Moreover, our results indicate an interplay between *Pseudomonas* MgtC role in macrophages and phagosome acidification.

### Expression of *P*. *aeruginosa mgtC* is induced within macrophages

**Fig 5:**
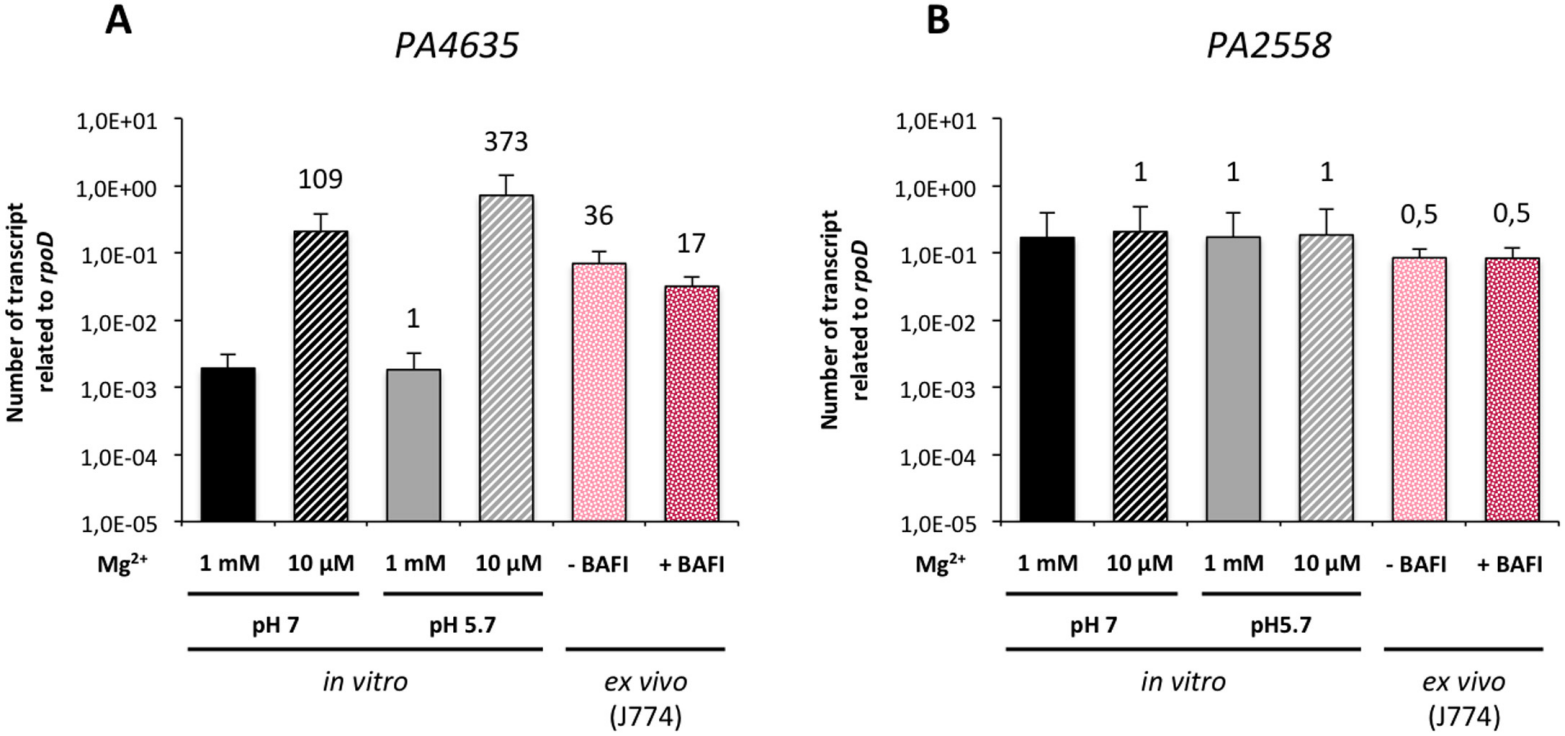
Expression of *P*. *aeruginosa mgtC* gene in liquid medium and intracellularly. The levels of *PA4635* (A) and *PA2558* (B) transcripts relative to those of the *rpoD* gene were measured by qRT-PCR. RNA was extracted from bacteria grown in liquid medium (*in vitro*) containing a high (1 mM) or low (10 μM) concentration of MgSO_4_ and a pH of 7 or at 5.7. A pH of 5.7 is expected to mimic the pH faced by bacteria in the phagosome. Bacterial RNA was also extracted from infected J774 macrophages (*ex vivo*) that were treated (+ BAFI) or not (- BAFI) with bafilomycin A1. For all conditions, RNA were prepared two times independently. Results are expressed as means ± SD from at least three independent measurements (each performed in triplicate). The numbers indicate the induction fold relatively to the condition 1 mM MgSO_4_ pH 7.

We first investigated the regulation of *mgtC* expression in liquid media depending on Mg^2+^ concentration and pH. Quantitative RT-PCR experiment showed that *mgtC* gene (*PA4635*) is strongly induced by low Mg^2+^ environment (Fig 5A). Moreover, in low Mg^2+^ condition, the level of *mgtC* gene expression can be further increased by lowering the pH (Fig 5A). On the other hand, the other *mgtC*-like gene (*PA2558*) was not regulated by Mg^2+^ nor pH (Fig 5B). We then quantified the *mgtC* mRNA level from infected macrophages. Importantly, we showed that *mgtC* is highly induced in macrophages, which agrees with a specific role in this step. On the other hand *PA2558*, as well as the T3SS gene *pcrV* or the house-keeping gene *gyrA* (S5 Fig), were similarly expressed in liquid medium and macrophages, and are therefore not induced in macrophages. Bafilomycin A1 treatment of macrophages, which decreases phagosome acidification, lowered *mgtC* expression intracellularly, which is consistent with the pH regulation observed *in vitro*. Taken together, these results indicate that *mgtC* is specifically expressed in macrophages and that phagosome acidification contributes to an optimal expression of the gene.

### Role of MgtC for *P*. *aeruginosa* growth in low magnesium medium and biofilm formation on abiotic surface

**Fig 6:**
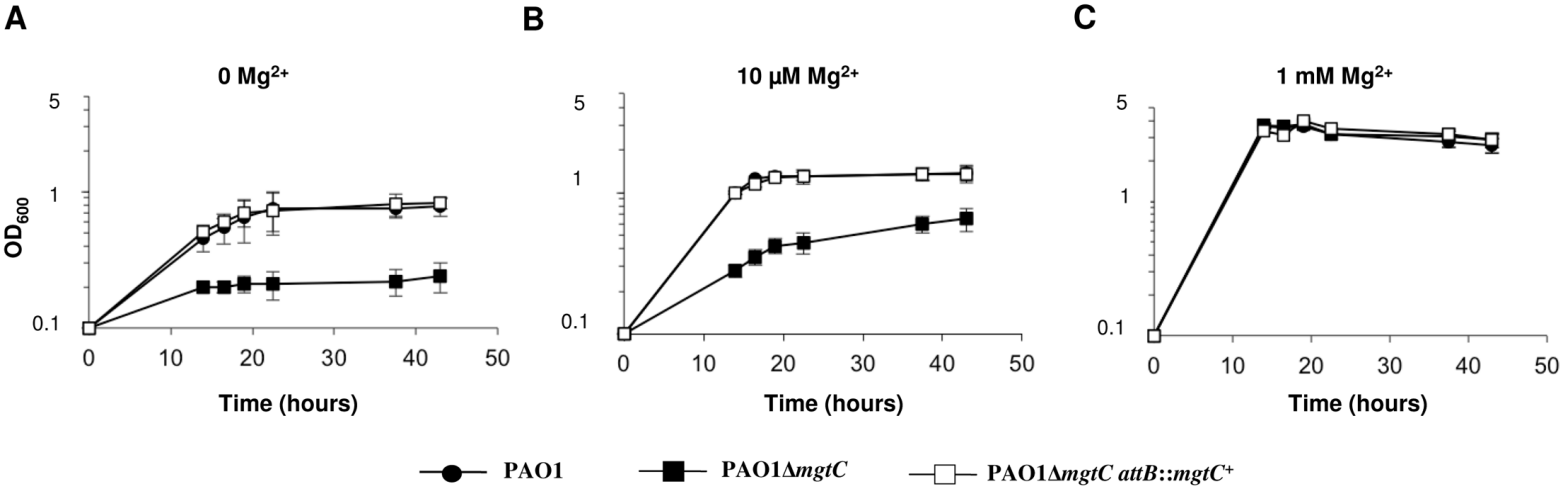
MgtC is required for growth of *P*. *aeruginosa* in Mg^2+^ deprived medium. (A) *P*. *aeruginosa* strains PAO1, PAO1Δ*mgtC*, and PAO1Δ*mgtC attB*::*mgtC* ^*+*^ were grown at 30°C in minimal medium without MgSO_4_. OD_600_ is indicated over the growth period. The experiment was independently repeated three times and results are expressed as means ± SD. Statistical analyses indicate significant difference between growth of the *mgtC* mutant and the wild-type or the complemented strain (Student’s t test, *P* <0.001 at times 20 hrs and 40 hrs). (B) The same strains were grown in medium with 10 μM MgSO_4_. Statistical analyses indicate significant difference between growth of the *mgtC* mutant and the wild-type or the complemented strain (Student’s t test, *P* <0.001 at times 20 hrs and 40 hrs). (C) The same strains were grown in high magnesium medium (1 mM MgSO_4_). No significant difference is found between the three strains.

Bacterial growth of *P*. *aeruginosa* strains was measured in minimal medium deprived for magnesium (without added Mg^2+^ or with 10 μM Mg^2+^) or containing 1 mM Mg^2+^ (corresponding to physiological concentration). Growth of the *mgtC* mutant was significantly impaired in low Mg^2+^ media (Fig 6A and Fig 6B), while it did grow similarly to the PAO1 wild-type strain in high Mg^2+^ medium (Fig 6C). The complemented strain displayed a wild-type growth in these conditions. Hence, MgtC contributes to the adaptation of *P*. *aeruginosa* to low Mg^2+^ environment, similarly to MgtC homologues from intracellular pathogens [1]. This result is consistent with the induction of *mgtC* gene expression in low Mg^2+^ environment (Fig 5A). To test whether the phenotype observed with the *mgtC* mutant in macrophages may be related to the growth defect upon Mg^2+^ limitation, the killing assay by J774 macrophages was repeated upon addition of 25 mM Mg^2+^ in the DMEM medium. However, addition of Mg^2+^ did not rescue the enhanced sensitivity of the *mgtC* mutant to macrophages (S6 Fig), suggesting that the increased killing of the mutant is not related to intracellular Mg^2+^ limiting environment.

**Fig 7:**
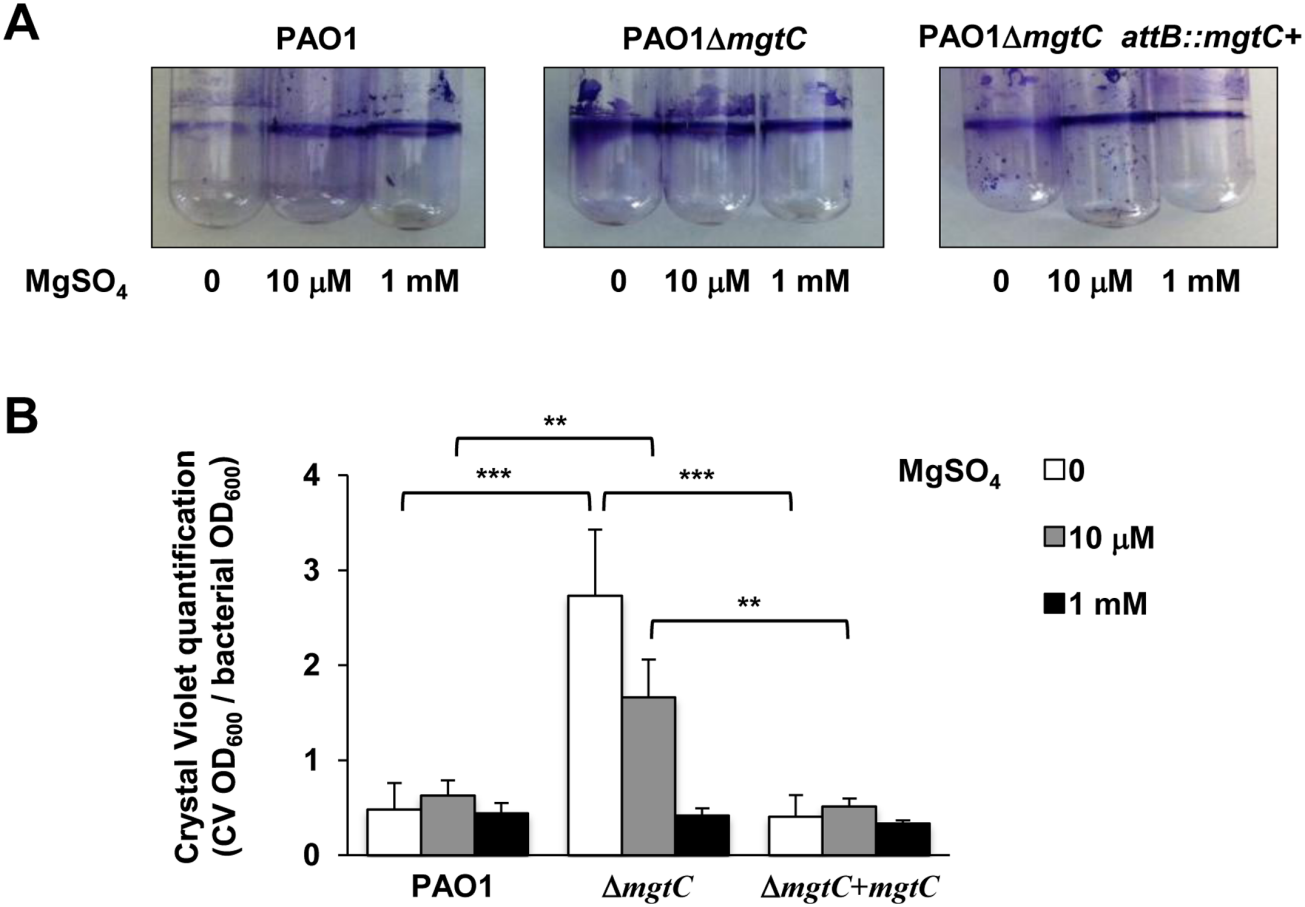
Quantification of bacterial adherence to glass tubes to infer the ability of strains to form biofilm. (A) Adherence assay with *P*. *aeruginosa* strains PAO1, PAO1Δ*mgtC*, and PAO1Δ*mgtC attB*::*mgtC* ^*+*^ grown at 30°C for 24 hrs in minimal medium without MgSO_4_ or with MgSO_4_ (10 μM and 1 mM). The biofilm quantification is visualized by crystal violet ring on the glass tube. (B) Crystal violet quantification (CV OD_600_) is divided by the bacterial density (bacterial OD_600_). Error bars correspond to standard errors (+ SE) from three independent experiments and the asterisks indicate *P* values (Student’s t test, **P <0.01, ***P <0.001).

*P*. *aeruginosa* ability to form biofilm is a crucial virulence determinant, more particularly in chronic infection. Interestingly, biofilm formation has been shown to be sensitive to EDTA through chelation of divalent cations including Mg^2+^ [18]. To check the role of both MgtC and Mg^2+^ ions during biofilm formation, wild-type, mutant and complemented strains were grown in different Mg^2+^ concentrations in glass tubes for 24 h and bacterial adherence to the glass was visualized (Fig 7A) and quantified using crystal violet staining (Fig 7B) to infer the ability of strains to form biofilm. Profiles of the three strains were the same in both assays if bacteria are grown in high Mg^2+^, indicating similar biofilm formation at physiological Mg^2+^ concentration. However, in the absence of Mg^2+^, wild-type and complemented strains poorly attached to the glass, indicating that Mg^2+^ is required for biofilm initiation by *P*. *aeruginosa*. In contrast, the Δ*mgtC* mutant was still able to form biofilm. The increased biofilm formation for the Δ*mgtC* mutant comparatively to the wild-type strain was also observed in the presence of 10 μM Mg^2+^. Cumulatively, these results indicate that when expressed, i.e. under Mg^2+^ limitation, MgtC limits biofilm formation. Since exopolysaccharides (EPS) are essential biofilm matrix components, important for initial adherence and biofilm formation [32,33], EPS production was measured by Congo red assay [34] in low and high Mg^2+^ (S7 Fig). Whereas, all strains produced similar EPS level at 1 mM Mg^2+^, the mutant strain produced significantly more EPS than wild-type and complemented strain in low Mg^2+^ medium.

We also investigated whether the increased biofilm formation of the *mgtC* mutant strain under Mg^2+^ limitation may be related to altered properties of the flagellum or the type IV pili, which are major adhesins required for initial attachment [35]. Our results indicated that the twitching and swimming motilities, which depend on type IV pili and flagellum, respectively, were similar in wild-type, mutant and complemented strain (S8 Fig). On the other hand, we observed that *P*. *aeruginosa* Δ*mgtC* bacteria grown under Mg^2+^starvation were more elongated than wild-type bacteria (S9 Fig). Hence the increased biofilm formation of the *mgtC* mutant strain under Mg^2+^ limitation may be due to increased EPS production and/or bacterial cell elongation.

### Heterologous production of the *Salmonella* MgtR peptide in PAO1 mimics the *mgtC* mutant virulence phenotypes

**Fig 8:**
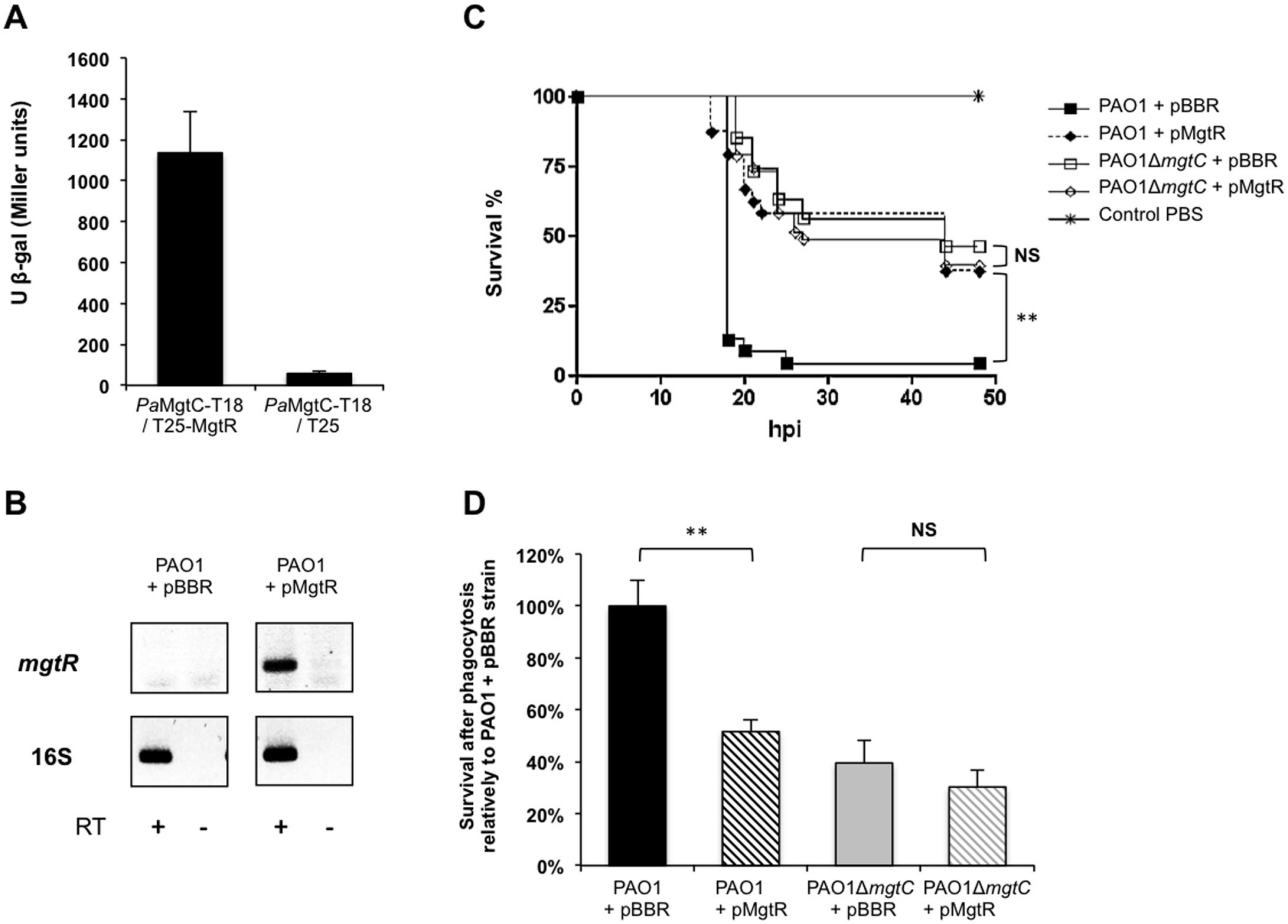
Virulence phenotypes of a PAO1 strain expressing *mgtR*. **(**A) *In vivo* interaction of *Pa* MgtC with MgtR peptide. The interaction was assayed using the BACTH system by transforming *E*. *coli* BTH101 cells with plasmids producing *Pa*MgtC-T18 and T25-MgtR. Liquid β-galactosidase assays were performed from six independent experiments. As negative control, BTH101 bacteria were cotransformed with a plasmid expressing *Pa*MgtC-T18 and the pKT25 vector. Error bars represent SD. (B) Expression of *Salmonella mgtR* in PAO1 strain. RT-PCR experiment was performed with primers specific for *mgtR* gene from RNA isolated from PAO1 strain carrying the pBBR1MCS vector (pBBR) or a pBBR1MCS derivative that encodes *mgtR* (pMgtR). Primers specific for 16S gene were used as positive control. Controls where reverse transcriptase was omitted are indicated (RT-). (C) Survival curve of embryos infected with PAO1 strain expressing or not *mgtR*. PAO1Δ*mgtC* strain expressing or not *mgtR* is also included in the experiment and non-injected embryos were used as control. Approximately 600–1000 CFU of *P*. *aeruginosa* were microinjected into the caudal vein (n = 24 per group). Results are expressed as the percentage of surviving on hour post-infection (hpi). Representative results of at least three biologically independent replicates are shown. Embryos are significantly more resistant to infection with PAO1 strain expressing *mgtR* than PAO1 strain with empty vector (*P*<0.01). (D) Behaviour of the PAO1 strain expressing *mgtR* in J774 macrophages. PAO1Δ*mgtC* strain expressing or not *mgtR* is included in the experiment. Results are normalized to 100% for the PAO1-pBBR strain and are expressed as means +SE from four independent experiments. Asterisks indicate statistical significance ** *P* <0.01). NS, non significant.

MgtR is a membrane peptide (30 amino-acid long) encoded downstream of *mgtC* in *S*. Typhimurium that regulates negatively MgtC expression [8]. MgtR interacts directly with *Salmonella* MgtC in a bacterial two-hybrid system and over-expression of *mgtR* in a wild-type *Salmonella* strain reduced significantly the ability of the strain to grow within macrophages, thus displaying an anti-infective effect [8]. Site-directed mutagenesis and structural analysis have suggested that MgtR interacts with the 4th transmembrane domain of MgtC [8,36]. Examination of the DNA region adjacent to *PA4635* as well as Blast analyses did not reveal any peptide similar to MgtR in *P*. *aeruginosa*. Because the 4th transmembrane domain of MgtC is well conserved between *S*. Typhimurium and *P*. *aeruginosa* (S10 Fig), we have tested whether the *Salmonella* MgtR peptide could interact with *P*. *aeruginosa* MgtC (*Pa*MgtC) using the bacterial two-hybrid system BACTH [37]. The interaction between T18-*Pa*MgtC and T25-MgtR fusion proteins was evaluated by measuring β-galactosidase activity (Fig 8A). Negative controls consisted in T18-*Pa*MgtC with empty pKT25 (Fig 8A) and empty pUT18 with T25-MgtR (S11 Fig). The high level of β-galactosidase activity, which was 60 fold the value of the negative control, was indicative of an interaction between the MgtR peptide and *Pa*MgtC in this system. On the other hand, *Pa*MgtC did not interact with another membrane peptide, the *Salmonella* KdpF peptide (S11 Fig), indicating that the interaction with MgtR is not due to sticky properties of *Pa*MgtC.

We then transformed the wild-type *P*. *aeruginosa* strain with pMgtR, a plasmid that harbors the *Salmonella mgtR* gene [8]. Expression of *mgtR* in PAO1 was verified by RT-PCR (Fig 8B). The virulence of the strain was evaluated using the zebrafish embryo model. As shown in Fig 8C, a wild-type PAO1 strain that expressed *mgtR* was attenuated comparatively to a control PAO1 strain that harbored an empty vector. On the other hand, no effect of the pMgtR plasmid was detected in the context of the PAO1 *mgtC* mutant strain. Expression of *mgtR* also increased significantly the killing of wild-type PAO1 strain, whereas no significant effect was found in the presence of the *mgtC* mutation (Fig 8D). In addition, a mild but significant increase in phagocytosis was observed (S12 Fig). Taken together, these results suggest that heterologous production of the *Salmonella* MgtR peptide can lower PAO1 virulence and resistance to macrophage killing by targeting the *P*. *aeruginosa* MgtC. Regarding other phenotypes related to MgtC defect in *P*. *aeruginosa*, production of the *Salmonella* MgtR peptide did not modulate bacterial growth in magnesium deprived medium (S13 Fig). This correlates with results obtained in *Salmonella* and is consistent with the finding of a dual role for MgtC [15].

## Discussion

**Fig 9:**
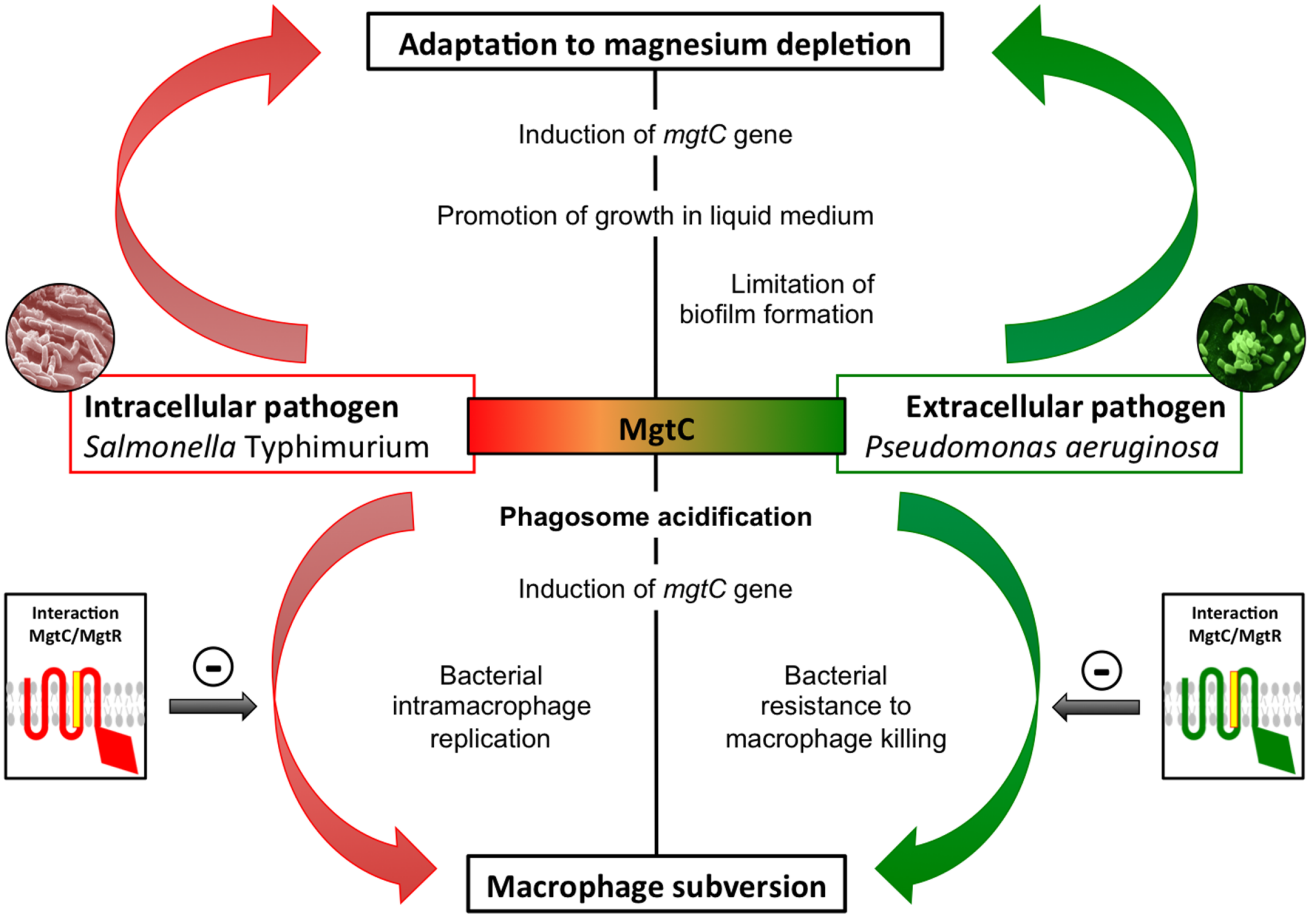
Role of MgtC in intracellular and extracellular pathogens. MgtC is involved in adaptation to magnesium deprivation and macrophage subversion both in intracellular and extracellular pathogens. We propose that MgtC share a similar function in intracellular and extracellular pathogens and that the bacterial lifestyle determines whether MgtC will promote intramacrophage bacterial multiplication or resistance to killing by macrophages. Expression of *mgtC* gene is induced in macrophages and phagosome acidification contributes to its optimal expression. The MgtR peptide can interfere with MgtC function, thus acting as a natural antagonist, and its production limits the macrophage subversion.

Phylogenetic analysis indicated that the MgtC protein from the extracellular pathogen *P*. *aeruginosa* clustered in the same subgroup as MgtC proteins from intracellular pathogens, which are known to subvert macrophages and promote intracellular bacterial multiplication [1,16]. MgtC exhibits a sporadic distribution in *Pseudomonas* species, being mostly associated with strains that are pathogenic for humans and insects. In the present study, we show that MgtC plays a role in *P*. *aeruginosa* virulence in a systemic model of infection in a macrophage-dependent manner and in *P*. *aeruginosa* ability to escape the killing action of the macrophage. MgtC thus provides a singular example of a virulence determinant that subverts the antimicrobial behavior of macrophages both in intracellular and extracellular pathogens (Fig 9).

There is growing evidence that some cytotoxic extracellular pathogens can actually survive inside macrophages after phagocytosis to hide from the immune system [38], but little is known about the intracellular behavior of these pathogens. The zebrafish embryo model has contributed significantly to the study of an intracellular phase in phagocytic cells in the life cycle of *Staphylococcus aureus*, a pathogen that has long been considered as an extracellular pathogen [39]. Interestingly, MgtC has been shown to promote intramacrophage survival of *Yersinia*. *pestis* [12], which is a facultative intracellular pathogen that replicates mainly extracellularly and produces antiphagocytic factors. Our present results identify MgtC as a novel actor in *P*. *aeruginosa* virulence, playing a role in an intramacrophage phase of this extracellular pathogen. The ability of *P*. *aeruginosa* to inhibit the microbicidal mechanisms of phagocytic cells is much less documented than its cytotoxicity towards mammalian cells, which is linked to the production of exotoxins and injected effector proteins by the T3SS. We have shown that the *P*. *aeruginosa mgtC* mutant is attenuated for acute infection in zebrafish embryos, which possess professional phagocytes that can engulf and kill *P*. *aeruginosa* upon infection [20]. MgtC acts by protecting *P*. *aeruginosa* against phagocytes since macrophage depletion suppressed the difference between *mgtC* mutant and PAO1 strain. Interestingly, a T3SS mutant has been reported to have a similar behavior in zebrafish embryos [20]. However, the phenotypes of the two mutants differ in *ex vivo* experiments because, contrary to a T3SS mutant, the *mgtC* mutant is more sensitive than the wild-type strain to the bactericidal effect of J774 macrophages. In contrast, the effect of the *mgtC* mutant on cytotoxicity towards macrophages is minor relatively to the one of T3SS mutant.

Consistent with the role of *P*. *aeruginosa mgtC* within macrophages, expression of the gene is highly induced when the bacteria reside inside macrophages, which is not the case for the *mgtC*-like *PA2558* and for a T3SS gene. Strong intramacrophage induction has also been reported for the *Salmonella mgtC* gene [40] and to our knowledge, this is the first report of a *P*. *aeruginosa* gene induced within phagocytic cells. It has been proposed that phagosome acidification may contribute to *Salmonella* MgtC protein production via an increased level of cytoplasmic ATP [5,7]. Our results further support an interplay between MgtC role in macrophages and the function of the V-ATPase because inhibition of the V-ATPase suppressed the difference between wild-type strain and *mgtC* mutant strain. Noticeably, *Pseudomonas* wild-type strain, but not *mgtC* mutant strain, had a reduced macrophage survival in the presence of V-ATPase inhibitor. A reduced resistance of wild-type bacteria to macrophage killing upon bafilomycin treatment has been also described for intracellular bacteria, for which acidification is important for proper expression of genes required intracellularly [41,42]. We demonstrated that phagosome acidification contributes to an optimal production of *Pseudomonas mgtC*, which agrees with the maximization of *mgtC* induction *in vitro* at low pH. Cumulatively, our results identify *mgtC* as a key gene for *P*. *aeruginosa* intramacrophage stage. Moreover, uptake by macrophages, but not epithelial cells, was enhanced with the *P*. *aeruginosa mgtC* mutant compared to the wild-type strain. A similar phenotype has been described for a *M*. *marinum mgtC* mutant, with MgtC playing a role in later steps of the internalization process rather than initial attachment events of bacteria to macrophages [14]. Further experiments will be required to investigate the fate of intracellular bacteria but we propose that *P*. *aeruginosa* has acquired a macrophage subversion factor to resist to killing in case of phagocytosis by macrophages. Moreover, our findings highlight the importance of macrophage subversion by *P*. *aeruginosa* in a model of acute infection. In agreement, *mgtC* has been recovered in a recent screen as a *Pseudomonas* gene involved in bacterial fitness in a systemic model of infection in mice [43]. It would be of interest to further explore the role of *Pseudomonas* MgtC in the mice model, both during acute and chronic infection.

Growth kinetic analyses showed that MgtC is required for optimal growth in magnesium deprived medium in *P*. *aeruginosa*, which is a common feature with intracellular pathogens. This result agrees with previous complementation studies showing that heterologous expression of *P*. *aeruginosa mgtC* (*PA4635*) in a *S*. Typhimurium *mgtC* mutant restored the growth defect in Mg^2+^ deprived medium [15]. Moreover, we have shown that *mgtC* expression in *P*. *aeruginosa* is induced in conditions of Mg^2+^ starvation, which is consistent with a previous proteomic analysis [44], and implies a role of MgtC in such stressing condition. Hence, our results indicate a mechanism of convergent evolution between pathogens that have different niches in their ability to use MgtC to adapt to Mg^2+^ limitation (Fig 9). We believe that this ability does not account for *P*. *aeruginosa* MgtC role in macrophages because Mg^2+^ measurement in a phagosome indicated a concentration in the millimolar range [45] and because addition of extracellular Mg^2+^ did not rescue the increased sensitivity of *mgtC* mutant to macrophages.

Magnesium has been shown to influence attachment and subsequent *Pseudomonas* biofilm formation [46], which is a hallmark of *Pseudomonas* chronic infection, and biofilms are disrupted by cation chelators [18]. Moreover, Mg^2+^ is required for biofilm formation in fungal pathogens [47]. In Mg^2+^ limiting condition, the *P*. *aeruginosa mgtC* mutant retains the ability to form biofilm, while the wild-type strain is severely impaired for biofilm formation, indicating that MgtC limits biofilm formation. The biofilm phenotype of the *mgtC* mutant is associated with increased production of EPS, which are essential biofilm matrix components. In addition, biofilm formation could be favored by the elongated shape of the *mgtC* mutant grown under Mg^2+^ limitation, as bacterial elongation can promote biofilm formation [48]. Very interestingly, a recent report provides an unsuspected link between MgtC, cellulose (a polysaccharide associated with the formation of biofilm) and intramacrophage survival in *Salmonella* [49]. MgtC repressed cellulose biosynthesis during growth in low Mg^2+^ medium and cellulose production was shown to hinder *Salmonella* replication inside macrophages. Despite the fact that cellulose is not produced by *P*. *aeruginosa* strains (as PAO1), this new finding, combined to our finding of an increased EPS production by the *P*. *aeruginosa mgtC* mutant in low Mg^2+^ medium, suggests a potential link between EPS production and *P*. *aeruginosa* MgtC role in macrophages. Further studies will be required to determine the nature of the EPS involved, to investigate their production intracellularly and the putative link with intramacrophage phenotypes.

MgtC proteins consist of two structural domains, a highly conserved membrane N-terminal domain and a more divergent soluble C-terminal domain. Structural data as well as bioinformatic modeling indicate that the *P*. *aeruginosa* MgtC C-terminal domain adopts the same fold as the one of MgtC proteins from intracellular pathogens, and this despite a low level of sequence identity ([50]; G. Labesse, personal communication). This suggests that MgtC may share a similar function in *P*. *aeruginosa* and intracellular pathogens, which is supported by the common role in adaptation to Mg^2+^ deprivation. Furthermore, whereas expression of *P*. *aeruginosa mgtC* does not complement the macrophage replication defect of a *S*. Typhimurium *mgtC* mutant, a significant complementation is observed when a *P*. *aeruginosa* MgtC variant carrying a single amino-acid change is produced [15]. Hence, MgtC proteins from *P*. *aeruginosa* and *S*. Typhimurium may have evolved in a very subtle way, possibly to optimally interact with partner protein(s).

The MgtR membrane peptide has been proposed as a natural MgtC antagonist because in *S*. Typhimurium, the intramacrophage replication of a wild-type strain can be reduced upon over-production of MgtR, which plays a negative regulatory role on *mgtC* expression [8]. In the present study, we have found that the phenotypes observed with *Pseudomonas mgtC* mutant in animal and cellular infection models can be mimicked upon production of the *Salmonella* MgtR peptide in a wild-type *P*. *aeruginosa* strain. Experiments in *mgtC* mutant strain indicated that the peptide requires a functional MgtC protein to play a role. Hence, these results support the action of MgtR natural peptide as an antagonist of MgtC and highlight MgtC as a promising new target for anti-virulence strategies.

## Materials and Methods

### Bacterial strains and growth conditions

Bacterial strains and plasmids are described in S1 Table. *P*. *aeruginosa* was grown at 37°C in Luria broth (LB) or at 30°C in MM63 medium supplemented with 0.5% casamino acids, 0.2% glucose, and MgSO_4_ (10 μM or 1mM) or without MgSO_4_. Plasmids were introduced in *P*. *aeruginosa* by conjugation, using an *E*. *coli* strain containing pRK2013. Recombinant bacteria were selected on *Pseudomonas* isolation agar (PIA). *P*. *aeruginosa* mutants obtained by using the pKNG101 suicide vector were selected on plates containing 6% sucrose. Antibiotic concentrations (μg/ml) were: for *E*. *coli*: ampicillin, kanamycin, streptomycin, gentamicin, 50; tetracycline, 40; for *P*. *aeruginosa*: gentamicin, 50 or 125 (plates); tetracycline, 200; streptomycin, 2000.

### Construction of a *P*. *aeruginosa mgtC* mutant and complemented strain

To generate a non-polar deletion of the *mgtC* (*PA4635*) gene, 526 bp upstream and 580 bp downstream of the *mgtC* gene were amplified, cloned in pCR2.1 and subcloned in pKNG101 suicide vector giving the mutator pSBC44 which was mobilized in the wild-type *P*. *aeruginosa* strain PAO1 to select for double recombination event (S1 Text).

Complementation by a single copy of the *mgtC* ^+^ gene under its own promoter was carried out with the miniCTX integration method at *attB* region [51]. The entire *mgtC* gene with the 525 bp-upstream region, in which a σ^70^ dependent promoter was predicted (S1 Fig), was amplified, cloned in the pCR2.1 giving pSBC46, which was subcloned in mini-CTX1 plasmid vector giving pSBC47. Chromosomal insertion of pSBC47 carrying *mgtC* ^+^ and excision of plasmid DNA sequences resulted in an unmarked integrant (S1 Text).

### Infection of *Danio rerio* embryos

Experiments were performed using the *golden* zebrafish mutant [52] or the Tg(*mpeg1*::*mCherry*) [29] zebrafish line and maintained under standard conditions (supplemental material). Bacterial strains, which were freshly streaked out from glycerol stocks, were grown in LB medium to mid-log phase (DO = 0.6), recovered by centrifugation and washed twice in Phosphate-Buffered Saline (PBS). Suspensions were homogenized through a 26-gauge needle and resuspended in PBS at about 10^9^ bacteria/ml added with 10% phenol red to aid visualization of the injection process. Infections were carried out by microinjection of 1–2 nl of bacterial suspensions into the caudal vein of 30 hpf embryos, which were previously dechorionated and anesthetized with 0.02% tricaine. For survival kinetics after infection, the number of dead embryos was determined visually based on the absence of heartbeat. Depletion of macrophages was carried out upon microinjection of *pu*.*1* morpholinos into zebrafish eggs and visualized by fluorescence microscopy (S1 Text).

### Microscopic analysis of zebrafish embryos

For live imaging, anesthetized infected embryos were mounted in 35 mm dishes and immobilized with 1% low-melting point agarose. Direct visualization is performed using an Olympus MVX10 epifluorescent microscope equipped with a digital color camera (Olympus XC50). Fluorescence and bright-field images are acquired and processed with CellSens (Olympus) and assembled using GIMP 2.6 freeware to adjust levels and brightness and to remove out-of-focus background fluorescence.

For fixed samples observations, anesthetized embryos are fixed overnight at 4°C with 4% paraformaldehyde in PBS, then washed twice in PBS and transferred gradually from PBS to 50% glycerol-PBS. Fixed fishes were positioned flat onto depression transparent slides covered with a cover slip for observation with 40x Leica Apo oil 1.15 NA or 63x Leica Apo oil 1.33 NA objectives and using a Leica DM2500CSQ upright microscope equiped with a Leica TCS SPE confocal scan head and fluorescence and differential interference contrast (DIC) optics. Widefield fluorescence and DIC images were captured and assembled by LAS-AF software (Leica Microsystems).

### Ethics statement

All animal experiments described in the present study were conducted at the University Montpellier 2 according to European Union guidelines for handling of laboratory animals (http://ec.europa.eu/environment/chemicals/lab_animals/home_en.htm) and were approved by the Direction Sanitaire et Vétérinaire de l'Hérault and Comité d'Ethique pour l'Expérimentation Animale under reference CEEA-LR-13007. The breeding of adult fish adhered to the international guidelines specified by the EU Animal Protection Directive 2010/63/EU and adult zebrafish were not sacrificed for this study. All experiments were performed before the embryos free-feeding stage and did not fall under animal experimentation law according to the EU Animal Protection Directive 2010/63/EU. For survival curves, cardiac rhythm was used as a clinical criterion to fix the endpoint at which embryos are euthanized using the anaesthetic Tricaine up to a lethal dose (500 mg/ml). Embryos that survive infection were anaesthetized with Tricaine up to a lethal dose before bleach treatment.

### Cytotoxicity towards macrophages

The cytotoxicity of *P*. *aeruginosa* strains grown to mid-log phase in LB broth was assayed by using J774 macrophages as described in [53], except that macrophages were infected for 1.5 hr at a multiplicity of infection (MOI) of 20. The percentage of LDH release was calculated relatively to that of the uninfected control, which was set at 0% LDH release, and that of uninfected cells lysed with Triton X-100, which was set at 100% LDH release.

### Macrophage-mediated bactericidal assay

The killing of *P*. *aeruginosa* strains by J774 macrophages was carried out essentially as described previously [54]. Mid-log phase *P*. *aeruginosa* grown in LB broth was centrifuged and resuspended in PBS to infect J774 macrophages grown in DMEM medium supplemented with 10% FBS at a MOI of 10. After centrifugation of 24-well culture plates, bacterial phagocytosis was allowed to proceed for 30 min before washing three times with sterile PBS and adding fresh DMEM media supplemented with 400 μg ml^-1^ gentamicin. Macrophages were lysed after 1 h 30 min and 2 h 30 by using 1% Triton X-100 and the number of viable bacteria was determined by subsequent plating onto LB agar plates. The percentage of survival represents the ratio between the number of bacteria at time 1.5 hr or 2.5–3 hrs and the number of bacteria internalized after phagocytosis. In case of treatment with bafilomycin A1, the inhibitor was added at a concentration of 100 nM 30 min before phagocytosis and was also added after phagocytosis. Bafilomycin does not exhibit any bacterial toxicity at the working concentration. In experiments with strains expressing *mgtR*, macrophages were lysed after 20 min or 2 hrs of gentamicin treatment and the ratio between bacteria enumerated at the two time points was calculated.

### Analysis of intracellular bacteria by fluorescence microscopy

For analysis of bacteria in fixed cells, infection of J774 macrophages was carried out as described above for the CFU assay, except that macrophages were seeded on glass coverslips and infected at a MOI of 5 to 10 with bacteria that were previously passed four times through a 26G needle. Because fluorescent bacterial strains carry a plasmid-encoded gentamycin resistant gene, amikacin (300 μg ml^-1^) was used to kill extracellular bacteria. For time 0, cells have been in contact with amikacin for 20 min. At the end of the incubation period, the cells were fixed with 4% paraformaldehyde in PBS for at least 1 hr. Cells were washed three times with PBS and mounted on glass slides in Vectashield with DAPI (Vector Laboratories, Inc). The slides were examined using an upright fluorescence microscope (Axioimager Z2, Zeiss) equipped with an Apotome 1 for optical sectioning. A 63X Apochromat Objective (NA 1.4) was used, transmitted light was acquired using differential interference contrast (DIC), and m-Cherry fluorescence was acquired using a texas red filter set. Z-stacks were systematically acquired using a Z-step of 240 nm. Counting was performed taking into account a maximum projection of the Z-stack. Images were processed using ZEN blue software (Zeiss).

### RNA extraction and quantitative RT-PCR (qRT-PCR)

For the study of expression of *PA4635* and *PA2558* genes, RNA was prepared from mid-logarithmic bacterial cultures grown for 1 hour in NCE-minimal medium supplemented with 0.1% casamino acids, 38 mM glycerol and 10 μM MgSO_4_ or 1 mM MgSO_4_ [8] at pH 7 or pH 5.7. For *mgtR* expression study, RNA was prepared from mid-logarithmic bacterial cultures grown in LB. For bacterial RNA extraction from infected J774, 4.10^6^ macrophages were seeded into a 100 cm^2^ tissue culture dish and infected at an MOI of 10 as described above. One hour after phagocytosis, cells were washed three times with PBS, lysed with 0.1% Triton X100 and pelleted by centrifugation at 13 000 rpm for 10 min at 15°C. Bacteria were resuspended by adding 500 μl PBS and transferred to a new tube for RNA preparation (thus discarding non resuspended cellular debris).

RNA was prepared and reverse transcribed as previously described [55] except that bacteria were incubated only 5 min with RNA protect reagent and were disrupted with lyzozyme. Controls without reverse transcriptase were done on each RNA sample to rule out possible DNA contamination. Quantitative real-time PCR was performed using a Light Cycler 480 SYBR Green I Master mix and a 480 Light Cycler instrument (Roche). PCR conditions were as follows: 3 min denaturation at 98°C, 45 cycles of 98°C for 5 sec, 60°C for 10 sec and 72°C for 10 sec. PCR conditions for *mgtR* and 16S rRNA control gene were as follows: 25 cycles of 95°C for 30 sec, 46°C (59°C for 16S rRNA) for 20 sec and 72°C for 30 sec. The sequences of primers used for RT-PCR are listed in S1 Table.

### Internalization in non-phagocytic cells

Invasion assay of HeLa cells were done as described [22] with mid-log phase *P*. *aeruginosa* grown in LB broth.

### Adherence assay on inert surfaces and analysis of exopolysaccharide (EPS) production

The adherence assay was performed as previously described [56] except that the biofilm visualisation was done in glass tubes. O/N cultures were done in 2 ml of MM63 medium supplemented with glucose, Casamino acids and 1 mM MgSO_4_, cells were washed with PBS before resuspension in the same medium with various concentrations of MgSO_4_. The absorbance of the culture was measured at 600 nm (A_600_) before attached bacteria were stained with 0.1% crystal violet for a period of 15 min and washed twice with water. The stain was then dissolved in ethanol and absorbance was measured at 600 nm.

For liquid Congo red (CR) assays, bacteria were grown as previously in a minimal medium containing 40 μg/ml CR, under vigorous agitation. At 24 h, the A_600_ (absorbance at 600 nm) was determined and the bacterial cells were pelleted by centrifugation at 15493 g. For quantification of CR binding, the A_490_ of the supernatant of each sample was determined. The ratio of A_490_/A_600_ indicates the EPS production.

### Bacterial two-hybrid analysis

The *P*. *aeruginosa mgtC* gene, amplified using PAO1 chromosomal DNA and primers PA4635-pUT18-F/PA4635-pUT18-R, was cloned at the *Hind*III and *EcoR*I sites of the pUT18 vector, to produce a fusion protein *Pa*MgtC-T18. This recombinant plasmid was cotransformed with a plasmid producing a fusion protein T25-MgtR [8] into BTH101 bacteria. Quantification of interaction between fusion proteins was carried out at 30°C as described previously [8]. Values are average from at least six independent cultures. A level of β-galactosidase activity at least fivefold higher than that measured for vectors indicates an interaction.

### Statistical analysis

Statistical analyses of comparisons between survival curves were performed using the log rank test with Prism 4.0 (Graphpad, Inc). Statistical significance was assumed at *P* values <0.05. For *ex vivo* experiments (J774 or HeLa cells), paired Student’s t test was performed using Excel software (Microsoft). In the figures, * means *P* values <0.05, ** <0.01, and *** <0.001.

## Supporting Information

S1 Fig Construction of a *PA4635* mutant. The genomic organization of the *mgtC* (PA4635) locus and the sequence of a predicted σ70 promoter (BProm program (http://linux1.softberry.com/berry.phtml?topic=bprom&group=programs&subgroup=gfindb) found upstream *mgtC* are indicated. The position of oligonucleotides used in PCR experiment to check the mutant is shown.
(TIF)

S2 Fig Visualisation of macrophage depletion in zebrafish embryos. Depiction of a transgenic (*mpeg1*::*mCherry*) zebrafish embryo lacking macrophages generated by the injection of *pu1* morpholino mixture at one stage cell. The absence of macrophages in morphants was confirmed after an observation by fluorescent microscopy while the load of macrophages is normal in standard control (inset). Auto-fluorescence is visible at the yolk.
(TIF)

S3 Fig Survival curves of *pu*.*1* morphant embryos infected with a low bacterial dose (500 CFU). As shown by the graph, *pu*.*1* morphant embryos (n = 20 each) are sensitive to infection even at low bacterial dose (whereas non treated embryos survive when infected with such dose). No statistically significant difference is obtained between *pu*.*1* morphant embryos injected with PAO1 or Δ*mgtC* strain (ns: non significant).
(TIF)

S4 Fig MgtC is involved in uptake of *P*. *aeruginosa* by macrophages but not by non-phagocytic cells. (A) Phagocytosis of *P*. *aeruginosa* strains by J774 macrophages. A ratio is calculated between bacterial CFUs counted after phagocytosis and bacterial CFUs from the inoculum. The percentage of phagocytosis is normalized to the one of PAO1. All assays were performed a minimum of three times in triplicate. Error bars represent standard deviations and asterisk indicates *P* value (*P <0.05, ** *P* <0.01). (B) Standard bacterial invasion assays in HeLa cells upon infection with *P*. *aeruginosa* strains. A T6SS mutant (Δ*clpV2*) is included as negative control. The percentage of invasion of PAO1Δ*clpV2* or PAO1Δ*mgtC* is normalized to the one PAO1 (that represents an average of 2.5 x 10^4^ CFUs of internalized bacteria per well). All assays were performed a minimum of three times in triplicate. Error bars represent standard deviations and asterisk indicates *P* value (***P <0.001).
(TIF)

S5 Fig Expression of *P*. *aeruginosa pcrV and gyrA* genes in liquid medium and intracellularly The levels of *pcrV* (A) and *gyrA* (B) transcripts relative to those of the *rpoD* gene were measured by qRT-PCR. RNA was extracted from bacteria grown in liquid medium (*in vitro*) containing a high (1 mM) or low (10 μM) concentration of MgSO_4_ and a pH of 7 or at 5.7. Bacterial RNA was also extracted from infected J774 macrophages (*ex vivo*) that were treated (+ BAFI) or not (- BAFI) with bafilomycin A1. For all conditions, RNA were prepared two times independently. Results are expressed as means ±SD from at least three independent measurements (each performed in triplicate).
(TIF)

S6 Fig Effect of addition of extracellular magnesium on the survival of the Δ*mgtC* strain in J774 macrophages. The survival of bacteria was measured from macrophages cultured with or without additional magnesium (25 mM). Addition of extracellular magnesium does not rescue the survival defect of the Δ*mgtC* strain. The percentage of survival is normalized to the one of PAO1. Error bars correspond to standard errors (SE) from two independent experiments.
(TIF)

S7 Fig EPS production from *P*. *aeruginosa* strains. EPS production was measured by Congo Red staining from *P*. *aeruginosa* strains PAO1, PAO1Δ*mgtC*, and PAO1Δ*mgtC attB*::*mgtC* ^*+*^ grown at 30°C for 24 h in minimal medium with low MgSO_4_ (10 μM) or high MgSO_4_ (1 mM). Results are representatives of three independent experiments. Error bars correspond to standard errors (+ SE) from three independent experiments and the asterisks indicate *P* value (Student’s t test, ***P <0.001).
(TIF)

S8 Fig PAO1Δ*mgtC* mutant motility with twitching and swimming assays. *P*. *aeruginosa* strains PAO1, PAO1Δ*mgtC*, and PAO1Δ*mgtC attB*::*mgtC* ^*+*^ were grown in minimal medium containing 10 μM or 1 mM MgSO_4_. For the swimming assay, the particular aspect of colonies in 1 mM MgSO_4_ is not observed with the same MgSO_4_ concentration when the preculture is done in LB medium (lower panel) instead of minimal medium (MM).
(TIF)

S9 Fig PAO1Δ*mgtC* mutant morphology. *P*. *aeruginosa* strains PAO1, PAO1Δ*mgtC*, and PAO1Δ*mgtC attB*::*mgtC* ^*+*^ were grown at 30°C in minimal medium supplemented with 10 μM or 1 mM MgSO_4_. Strains were observed by optical microscopy at 100 X, Zeiss Axioskop 40. Scale bar indicates a length of 1 μm.
(TIF)

S10 Fig Alignment of amino-acid sequences of MgtC proteins from *S*. Typhimurium (STM3764) and *P*. *aeruginosa* (PA4635). Proteins were aligned using the ClustalX program. Conservation of residues is indicated below the sequences with the following rules: "*" for residues that are identical in all sequences in the alignment;":" for conserved substitutions; "." means for semi-conserved substitutions. Residues conserved in the phylogenetic subgroup only and subjected to site-directed mutagenesis in the present study are shaded in dark grey. Transmembrane (TM) domains are indicated above the sequence (TM1 to TM5). An Ala-coil motif, which is an helix-helix interaction motif characterized by small residues (Ala, Gly, Ser) in heptad repeats, present in the TM4 of *S*T MgtC (red rectangles) is conserved in PA4635. In addition, three residues important for *S*T MgtC/MgtR interaction between TM3 and TM4 are indicated by red dots.
(TIF)

S11 Fig *In vivo* interaction of *Pa*MgtC with *Salmonella* KdpF peptide. The interaction was assayed using the BACTH system by transforming *E*. *coli* BTH101 cells with plasmids producing *Pa*MgtC-T18 and T25-*St*KdpF. Liquid β-galactosidase assays were performed from three independent experiments in duplicate. As negative control, BTH101 bacteria were cotransformed with a plasmid expressing *Pa*MgtC-T18 and the pKT25 vector or with the pUT18 vector and a plasmid expressing T25-MgtR. Both negative controls give similar β-galactosidase levels. Transformants with *Pa*MgtC-T18 and T25-*St*KdpF give a β-galactosidase level that is not five times higher the level of negative controls, indicative of a lack of interaction between *Pa*MgtC and *St*KdpF according to the BACTH protocol. Error bars represent SD.
(TIF)

S12 Fig Phagocytosis of the PAO1 strain expressing *mgtR* by J774 macrophages. For PAO1 strain expressing or not *mgtR*, a ratio is calculated between bacterial CFUs counted after phagocytosis and 20 treatment of gentamycin and bacterial CFUs from the inoculum. Results are normalized to 100% for the PAO1-pBBR strain and are expressed as means +SE from four independent experiments. Asterisks indicate statistical significance * *P* <0.05).
(TIF)

S13 Fig Growth of *P*. *aeruginosa* PAO1 strain expressing *mgtR* in Mg^2+^ deprived medium. PAO1 strain carrying the pBBR1MCS vector (pBBR) or a pBBR1MCS derivative that encodes *mgtR* (pMgtR) were grown at 30°C in minimal medium with 0, 10 μM or 1 mM MgSO_4_. OD_600_ is indicated over the growth period. The experiment was independently repeated two times and a representative curve is shown.
(TIF)

S1 Table Bacterial strains, plasmids and primers used in the study. (DOC)

S1 Text Supporting Protocols. (DOC)
