# Supplementary figures and images for: A Macrophage Subversion Factor Is Shared by Intracellular and Extracellular Pathogens
Source: PLoS Pathog. 2015 Jun 16;11(6):e1004969. doi: 10.1371/journal.ppat.1004969 (PMC4469704; doi:10.1371/journal.ppat.1004969)

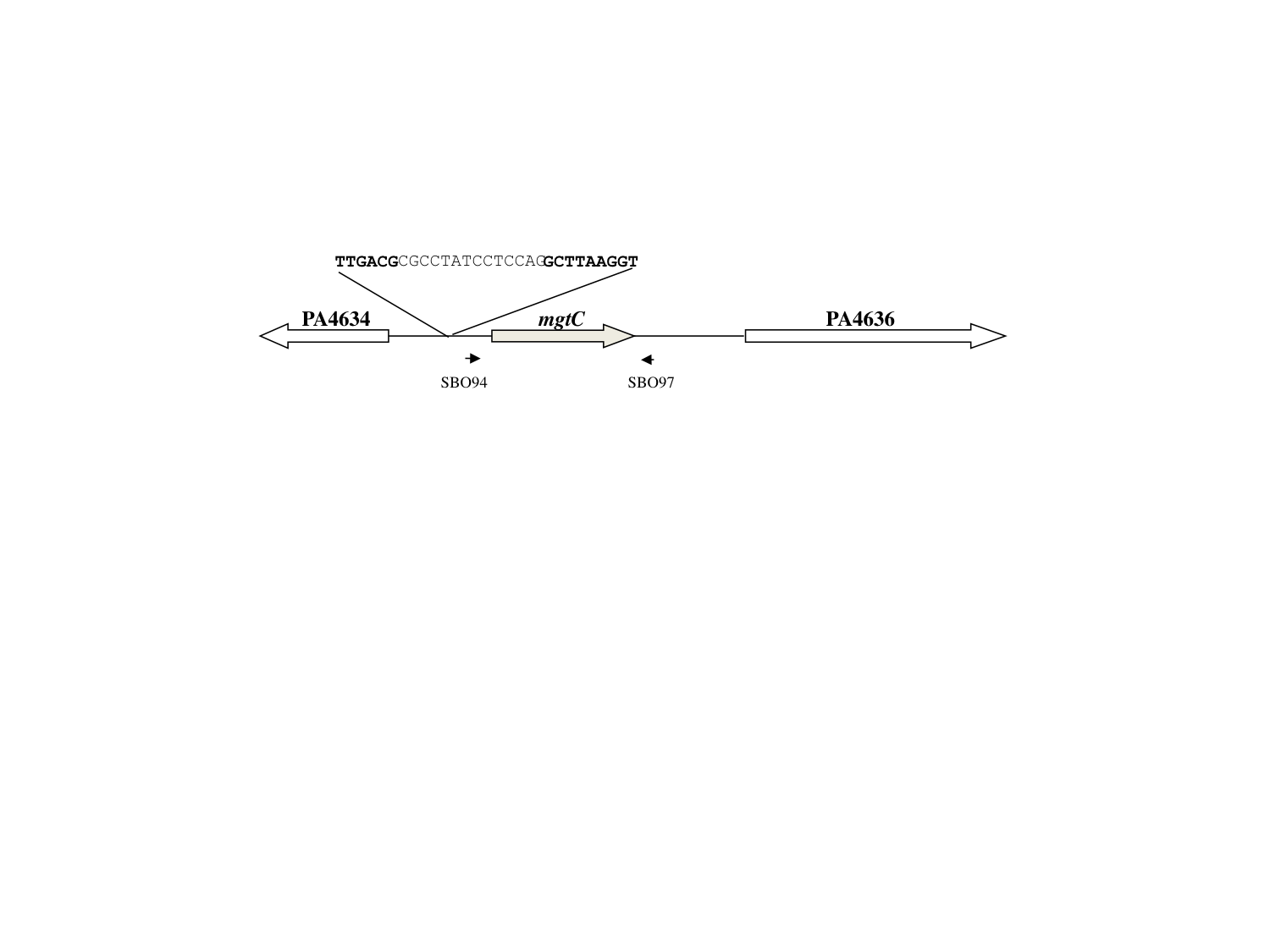

Supplement: S1 Fig — The genomic organization of the mgtC (PA4635) locus and the sequence of a predicted σ70 promoter (BProm program (http://linux1.softberry.com/berry.phtml?topic=bprom&group=programs&subgroup=gfindb) found upstream mgtC are indicated. The position of oligonucleotides used in PCR experiment to check the mutant is shown. (TIF) [file ppat.1004969.s001.tif]

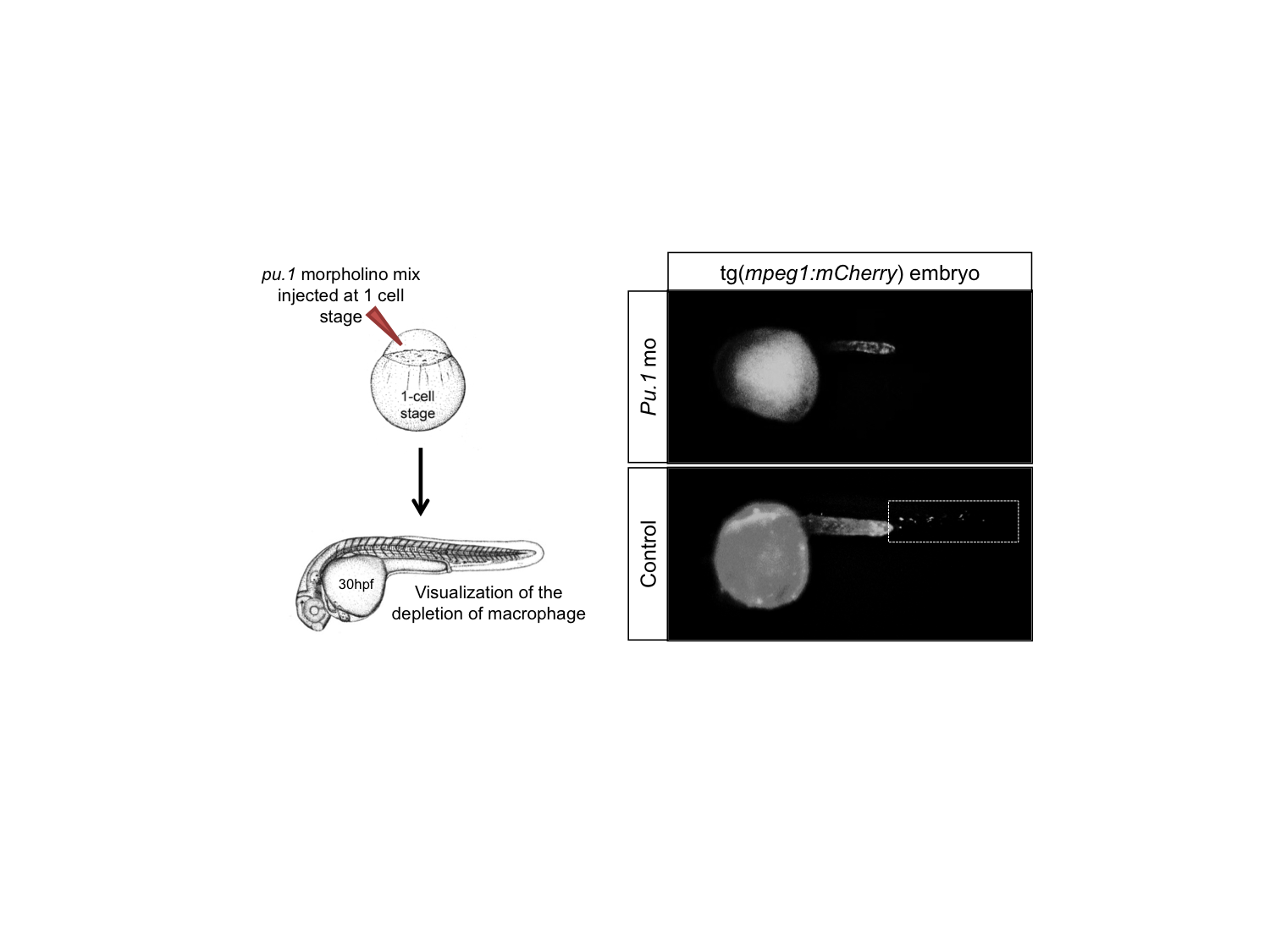

Supplement: S2 Fig — Depiction of a transgenic (mpeg1::mCherry) zebrafish embryo lacking macrophages generated by the injection of pu1 morpholino mixture at one stage cell. The absence of macrophages in morphants was confirmed after an observation by fluorescent microscopy while the load of macrophages is normal in standard control (inset). Auto-fluorescence is visible at the yolk. (TIF) [file ppat.1004969.s002.tif]

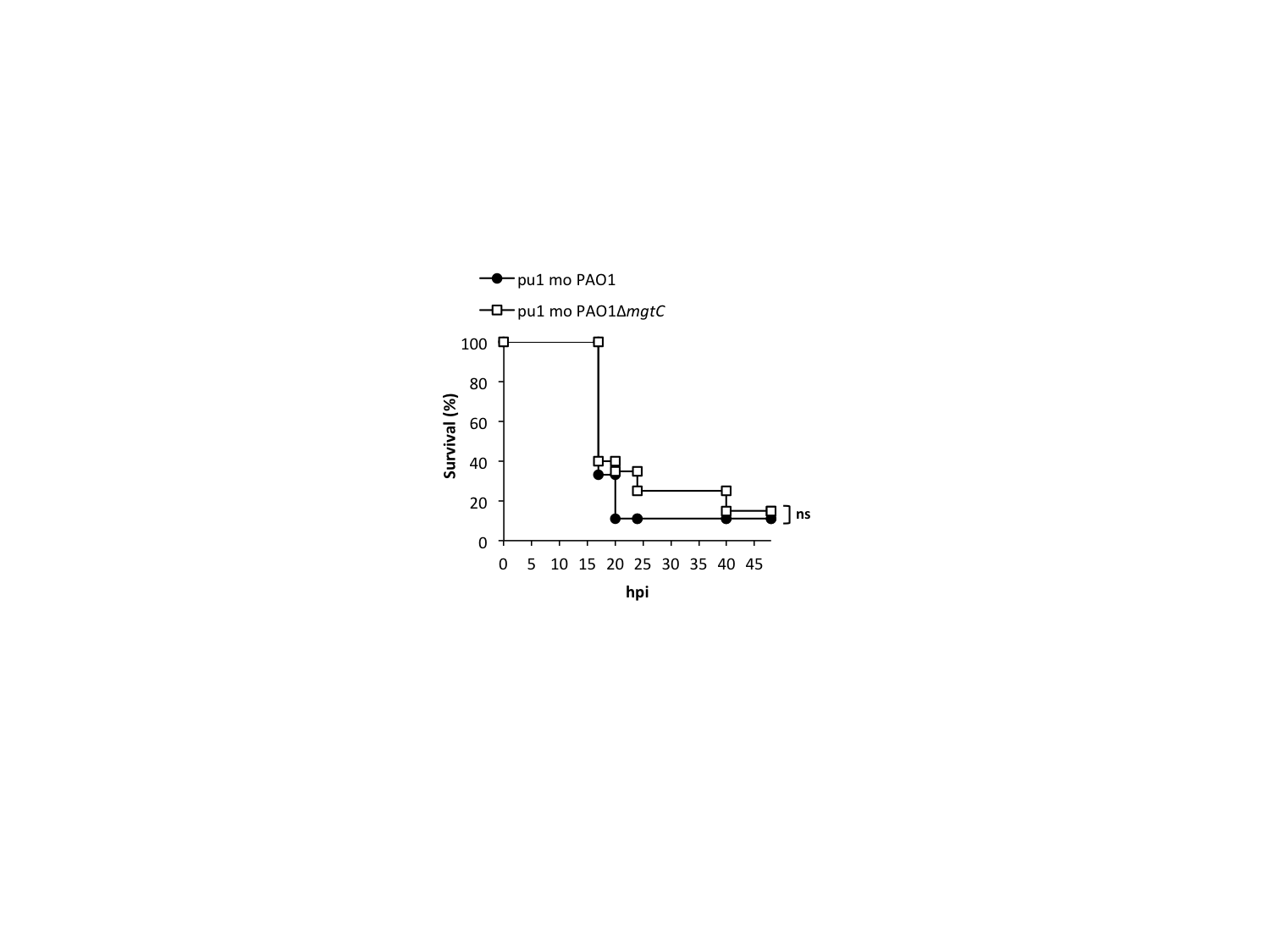

Supplement: S3 Fig — As shown by the graph, pu.1 morphant embryos (n = 20 each) are sensitive to infection even at low bacterial dose (whereas non treated embryos survive when infected with such dose). No statistically significant difference is obtained between pu.1 morphant embryos injected with PAO1 or ΔmgtC strain (ns: non significant). (TIF) [file ppat.1004969.s003.tif]

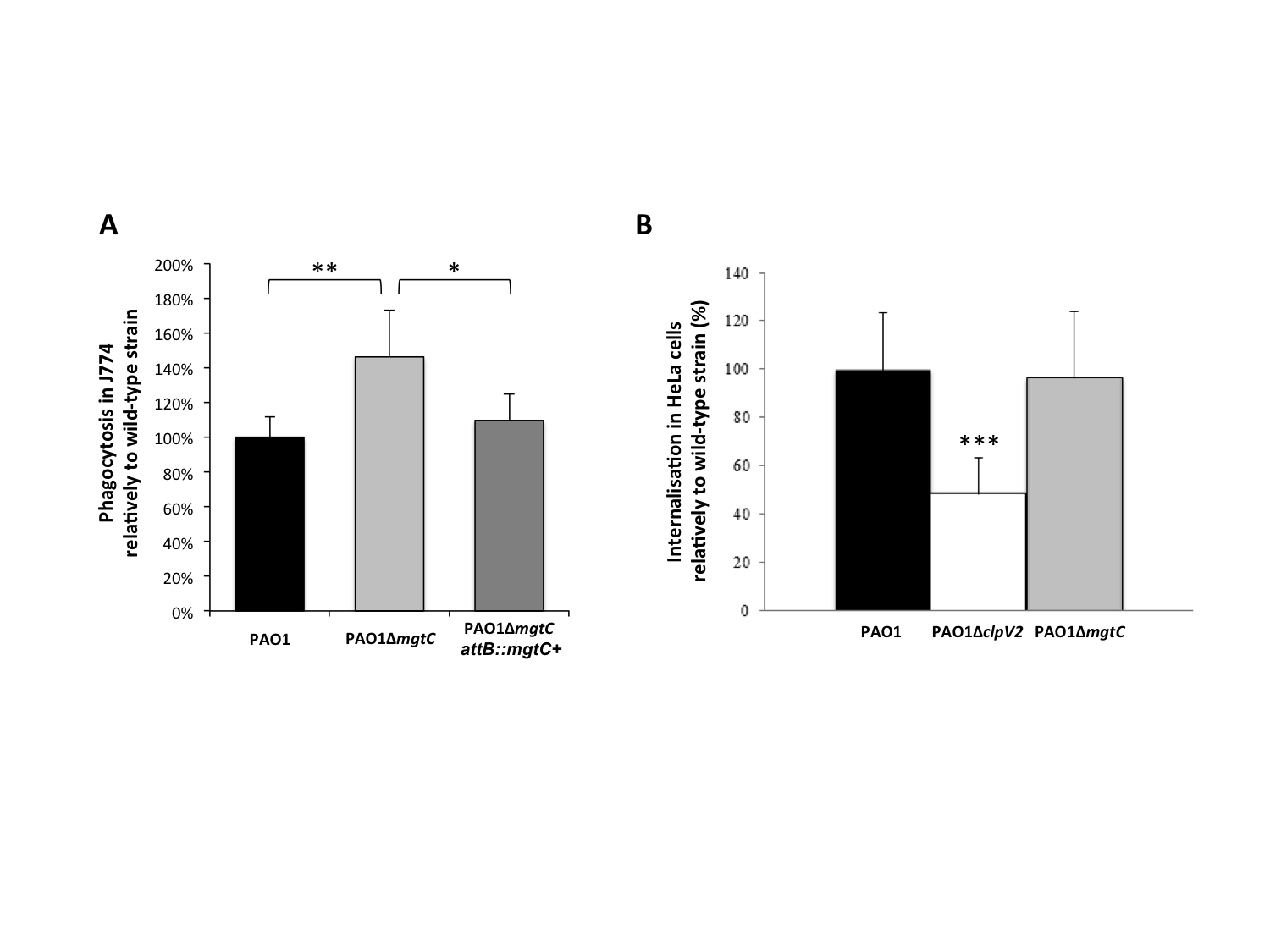

Supplement: S4 Fig — (A) Phagocytosis of P. aeruginosa strains by J774 macrophages. A ratio is calculated between bacterial CFUs counted after phagocytosis and bacterial CFUs from the inoculum. The percentage of phagocytosis is normalized to the one of PAO1. All assays were performed a minimum of three times in triplicate. Error bars represent standard deviations and asterisk indicates P value (*P <0.05, ** P <0.01). (B) Standard bacterial invasion assays in HeLa cells upon infection with P. aeruginosa strains. A T6SS mutant (ΔclpV2) is included as negative control. The percentage of invasion of PAO1ΔclpV2 or PAO1ΔmgtC is normalized to the one PAO1 (that represents an average of 2.5 x 10^4^ CFUs of internalized bacteria per well). All assays were performed a minimum of three times in triplicate. Error bars represent standard deviations and asterisk indicates P value (***P <0.001). (TIF) [file ppat.1004969.s004.tif]

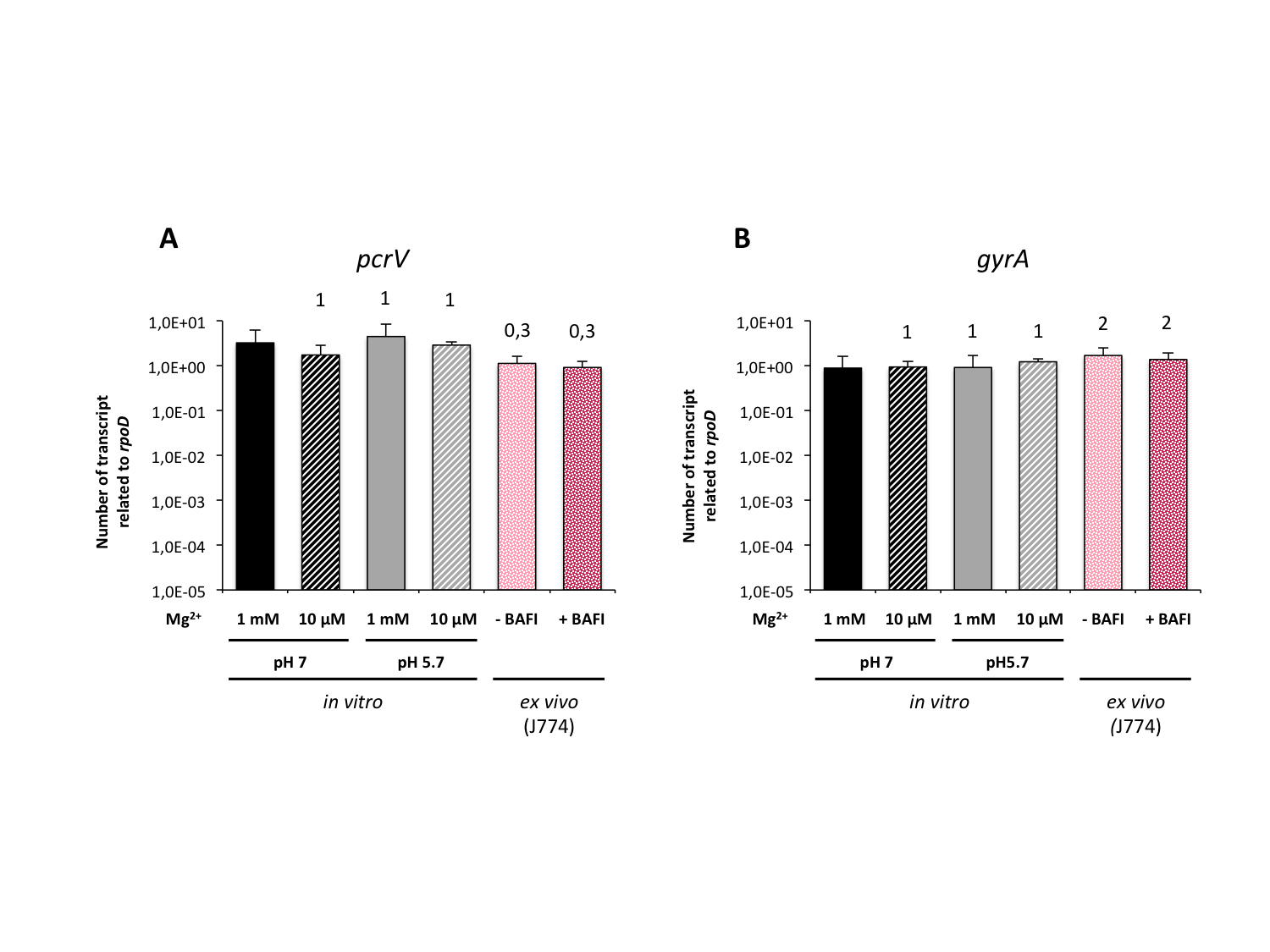

Supplement: S5 Fig — The levels of pcrV (A) and gyrA (B) transcripts relative to those of the rpoD gene were measured by qRT-PCR. RNA was extracted from bacteria grown in liquid medium (in vitro) containing a high (1 mM) or low (10 μM) concentration of MgSO4 and a pH of 7 or at 5.7. Bacterial RNA was also extracted from infected J774 macrophages (ex vivo) that were treated (+ BAFI) or not (- BAFI) with bafilomycin A1. For all conditions, RNA were prepared two times independently. Results are expressed as means ±SD from at least three independent measurements (each performed in triplicate). (TIF) [file ppat.1004969.s005.tif]

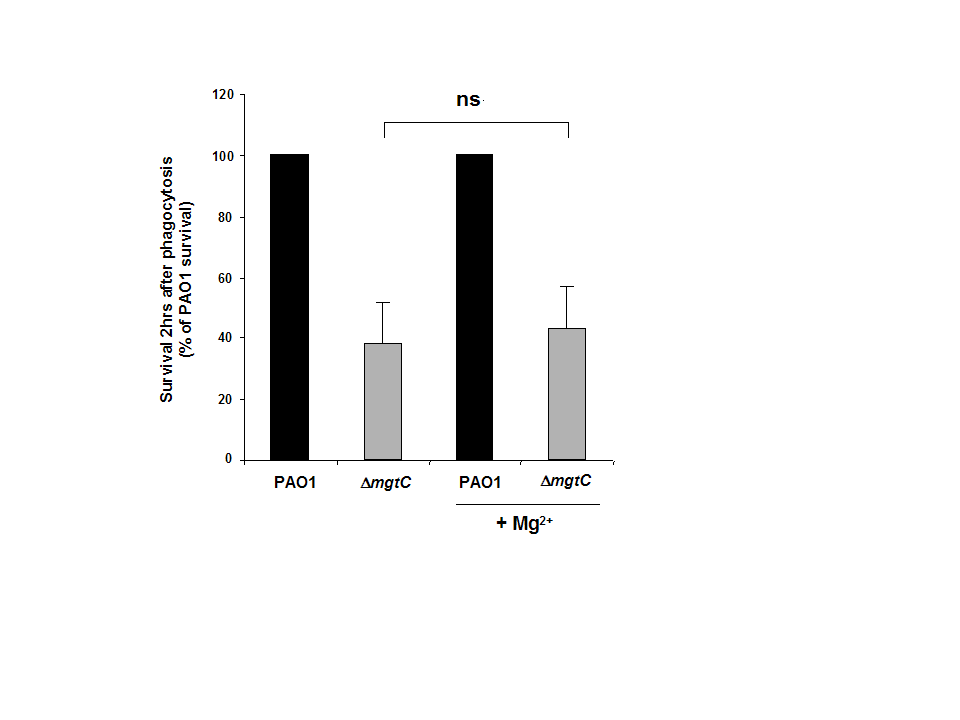

Supplement: S6 Fig — The survival of bacteria was measured from macrophages cultured with or without additional magnesium (25 mM). Addition of extracellular magnesium does not rescue the survival defect of the ΔmgtC strain. The percentage of survival is normalized to the one of PAO1. Error bars correspond to standard errors (SE) from two independent experiments. (TIF) [file ppat.1004969.s006.tif]

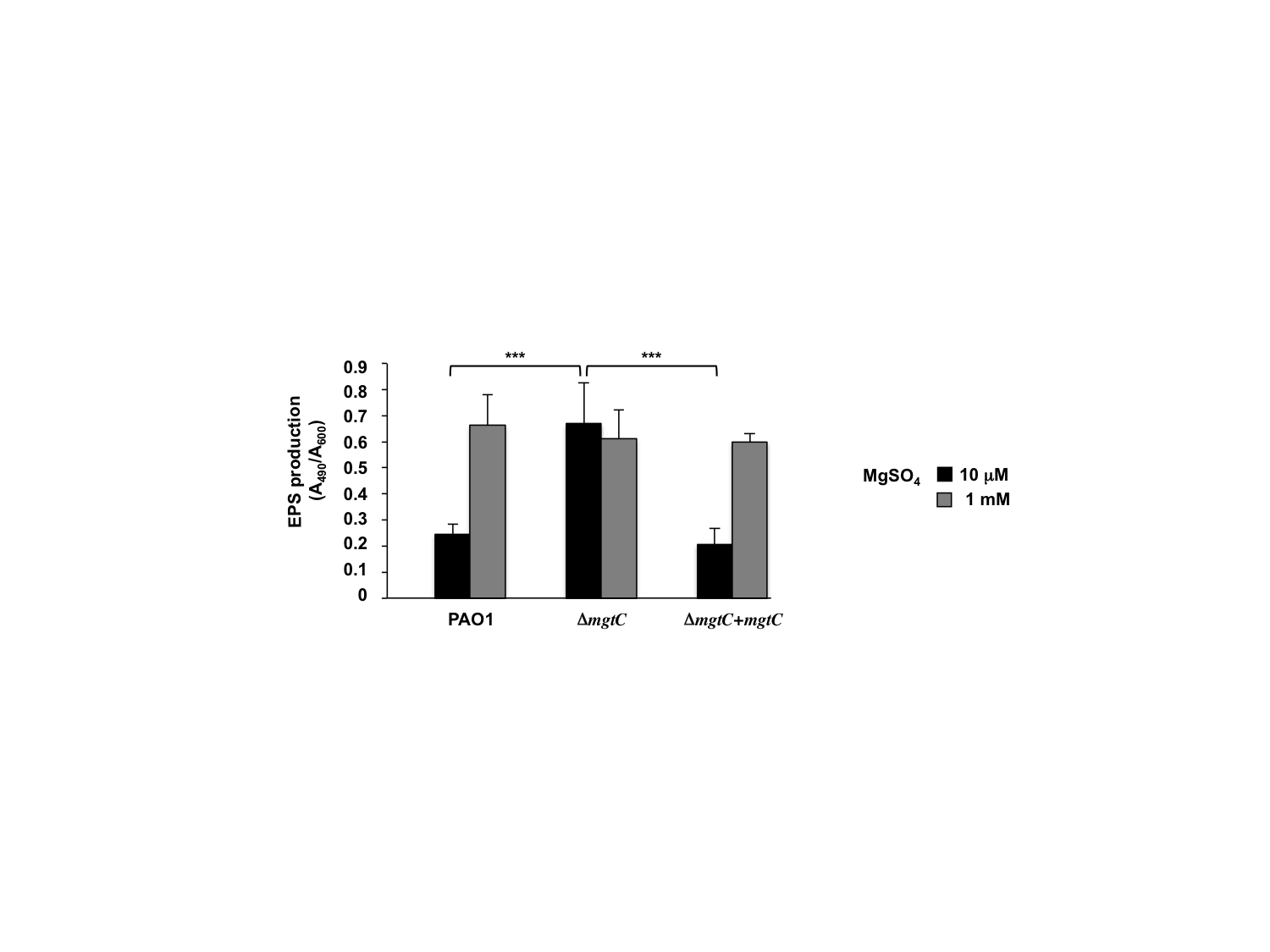

Supplement: S7 Fig — EPS production was measured by Congo Red staining from P. aeruginosa strains PAO1, PAO1ΔmgtC, and PAO1ΔmgtC attB::mgtC + grown at 30°C for 24 h in minimal medium with low MgSO4 (10 μM) or high MgSO4 (1 mM). Results are representatives of three independent experiments. Error bars correspond to standard errors (+ SE) from three independent experiments and the asterisks indicate P value (Student’s t test, ***P <0.001). (TIF) [file ppat.1004969.s007.tif]

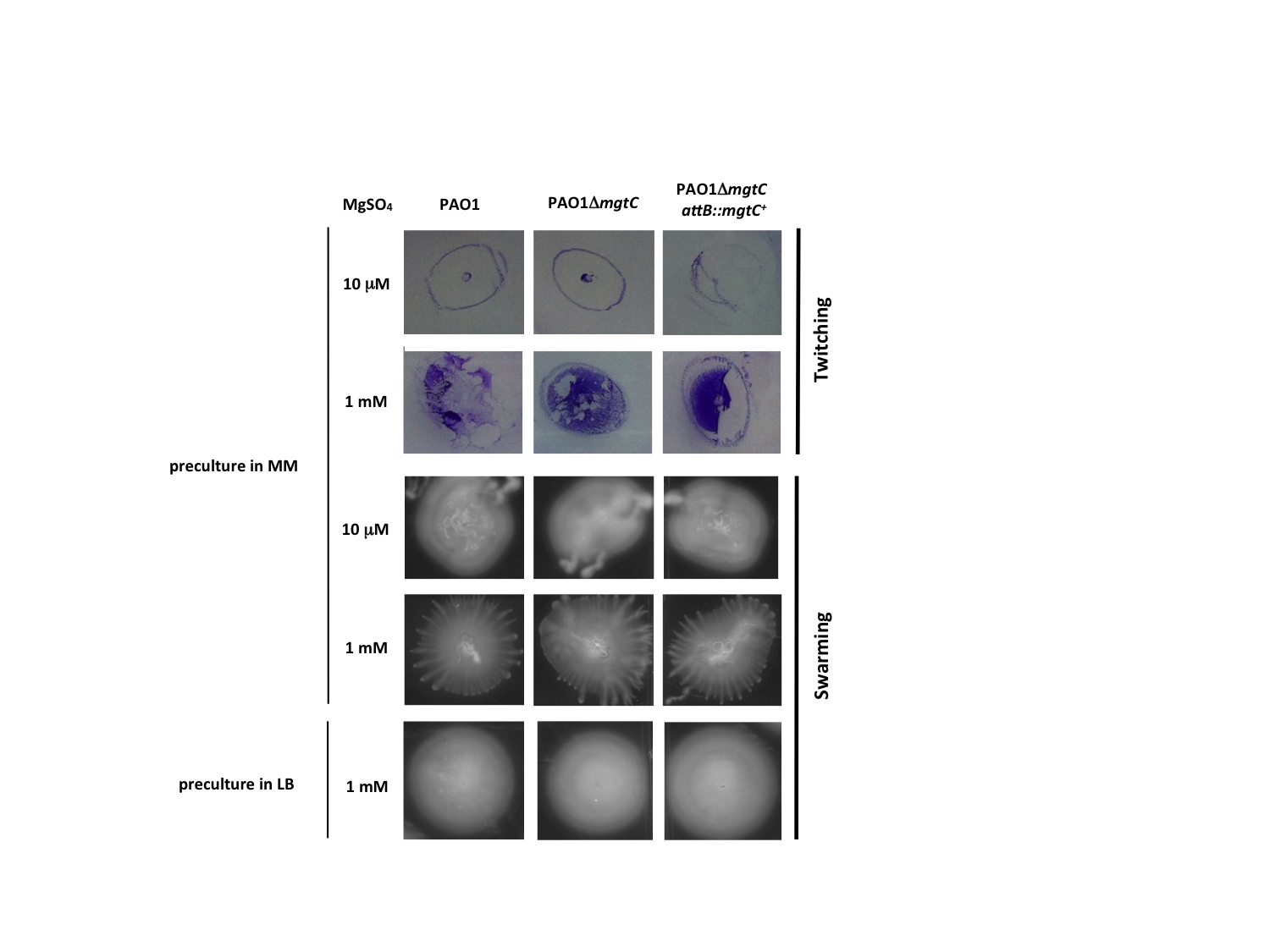

Supplement: S8 Fig — P. aeruginosa strains PAO1, PAO1ΔmgtC, and PAO1ΔmgtC attB::mgtC + were grown in minimal medium containing 10 μM or 1 mM MgSO4. For the swimming assay, the particular aspect of colonies in 1 mM MgSO4 is not observed with the same MgSO4 concentration when the preculture is done in LB medium (lower panel) instead of minimal medium (MM). (TIF) [file ppat.1004969.s008.tif]

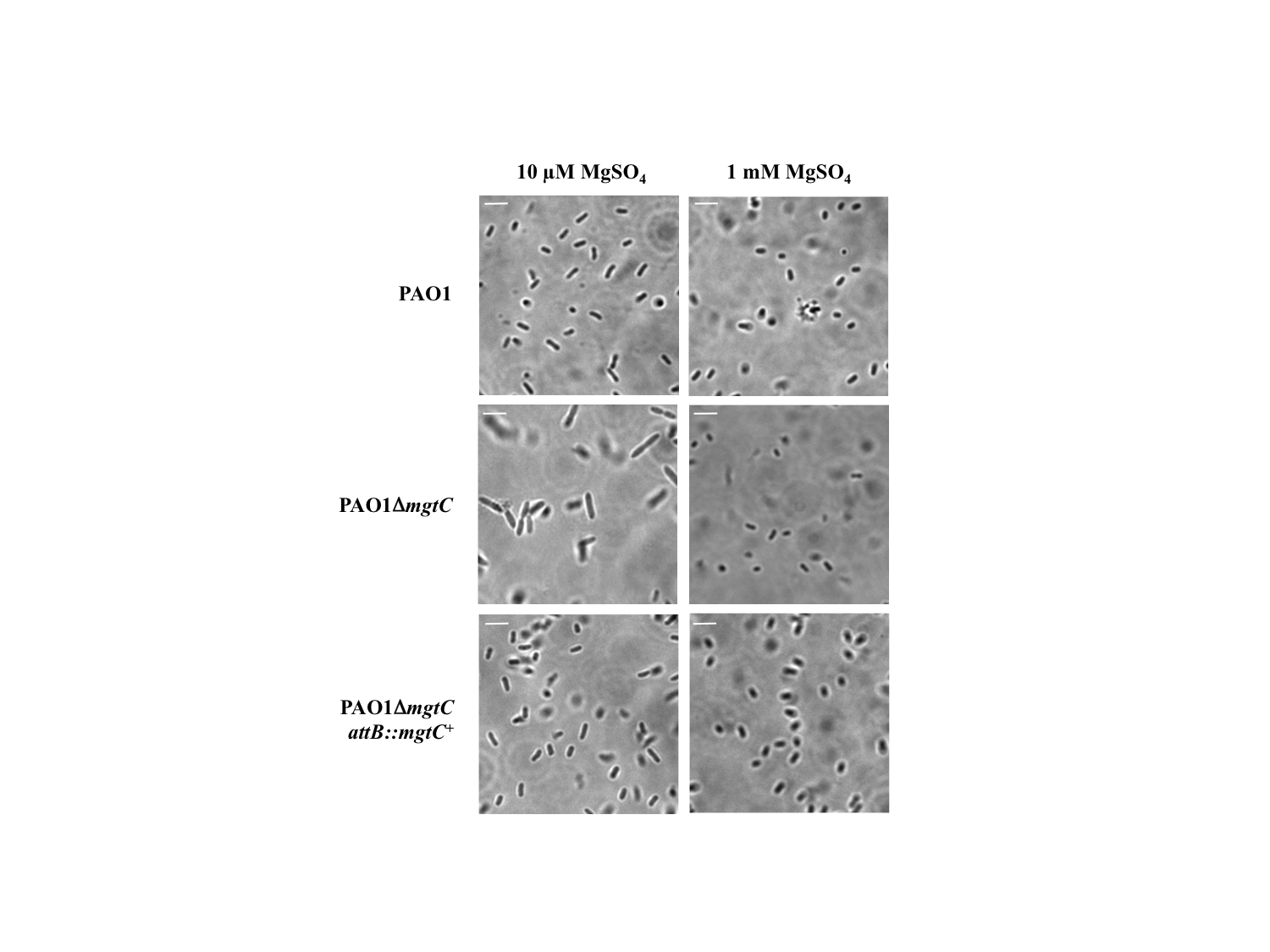

Supplement: S9 Fig — P. aeruginosa strains PAO1, PAO1ΔmgtC, and PAO1ΔmgtC attB::mgtC + were grown at 30°C in minimal medium supplemented with 10 μM or 1 mM MgSO4. Strains were observed by optical microscopy at 100 X, Zeiss Axioskop 40. Scale bar indicates a length of 1 μm. (TIF) [file ppat.1004969.s009.tif]

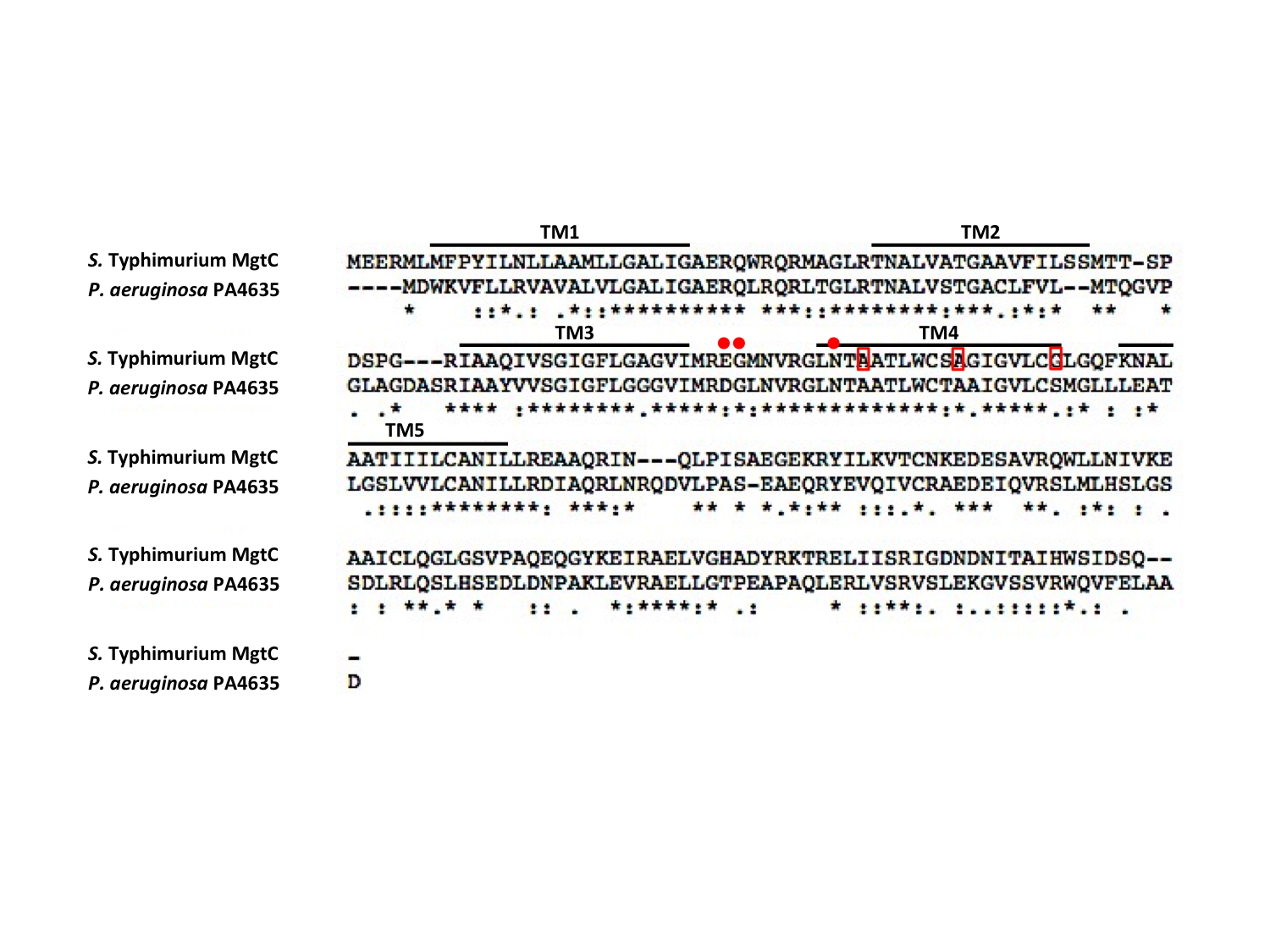

Supplement: S10 Fig — Proteins were aligned using the ClustalX program. Conservation of residues is indicated below the sequences with the following rules: "*" for residues that are identical in all sequences in the alignment;":" for conserved substitutions; "." means for semi-conserved substitutions. Residues conserved in the phylogenetic subgroup only and subjected to site-directed mutagenesis in the present study are shaded in dark grey. Transmembrane (TM) domains are indicated above the sequence (TM1 to TM5). An Ala-coil motif, which is an helix-helix interaction motif characterized by small residues (Ala, Gly, Ser) in heptad repeats, present in the TM4 of ST MgtC (red rectangles) is conserved in PA4635. In addition, three residues important for ST MgtC/MgtR interaction between TM3 and TM4 are indicated by red dots. (TIF) [file ppat.1004969.s010.tif]

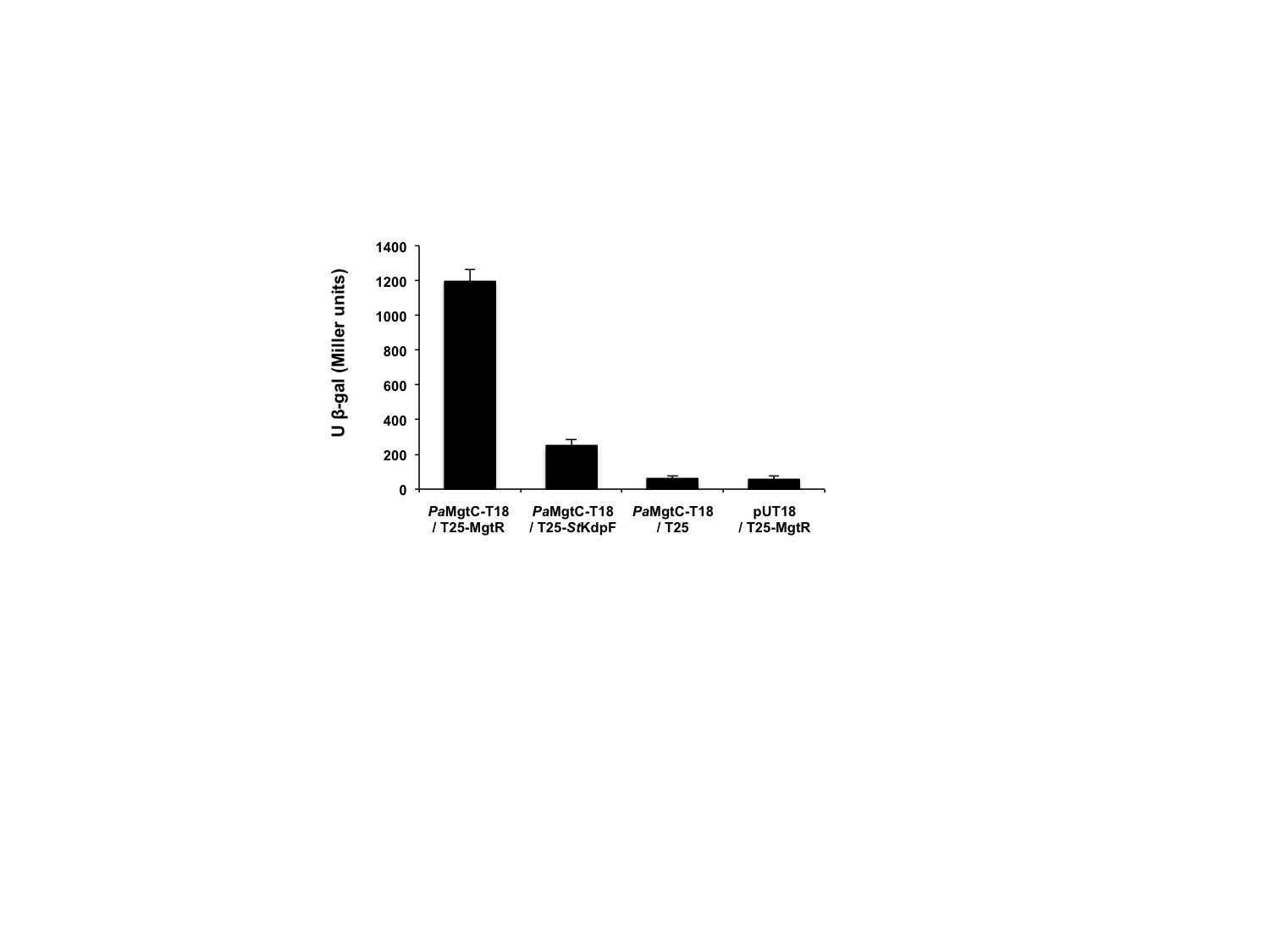

Supplement: S11 Fig — The interaction was assayed using the BACTH system by transforming E. coli BTH101 cells with plasmids producing PaMgtC-T18 and T25-StKdpF. Liquid β-galactosidase assays were performed from three independent experiments in duplicate. As negative control, BTH101 bacteria were cotransformed with a plasmid expressing PaMgtC-T18 and the pKT25 vector or with the pUT18 vector and a plasmid expressing T25-MgtR. Both negative controls give similar β-galactosidase levels. Transformants with PaMgtC-T18 and T25-StKdpF give a β-galactosidase level that is not five times higher the level of negative controls, indicative of a lack of interaction between PaMgtC and StKdpF according to the BACTH protocol. Error bars represent SD. (TIF) [file ppat.1004969.s011.tif]

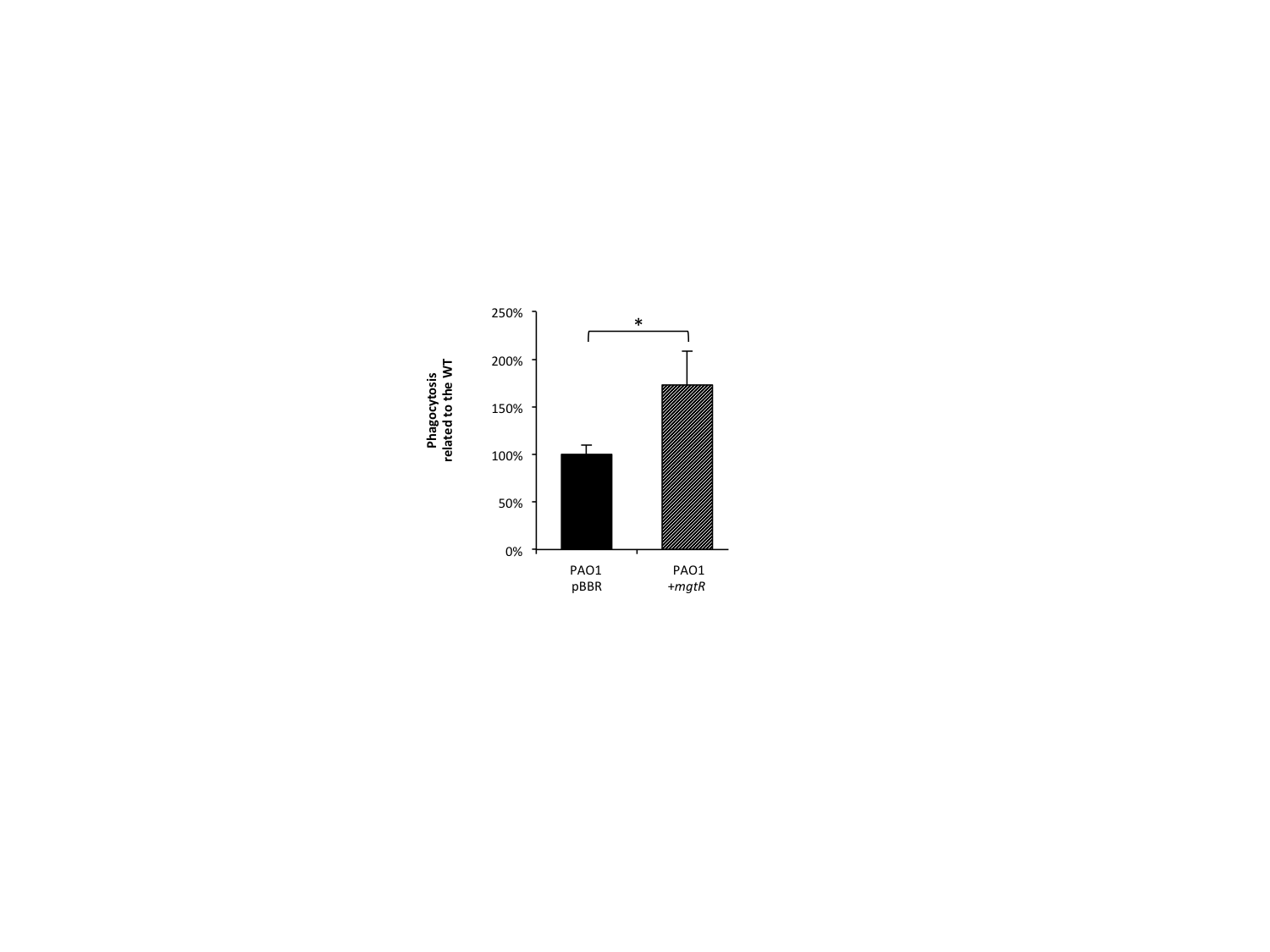

Supplement: S12 Fig — For PAO1 strain expressing or not mgtR, a ratio is calculated between bacterial CFUs counted after phagocytosis and 20 treatment of gentamycin and bacterial CFUs from the inoculum. Results are normalized to 100% for the PAO1-pBBR strain and are expressed as means +SE from four independent experiments. Asterisks indicate statistical significance * P <0.05). (TIF) [file ppat.1004969.s012.tif]

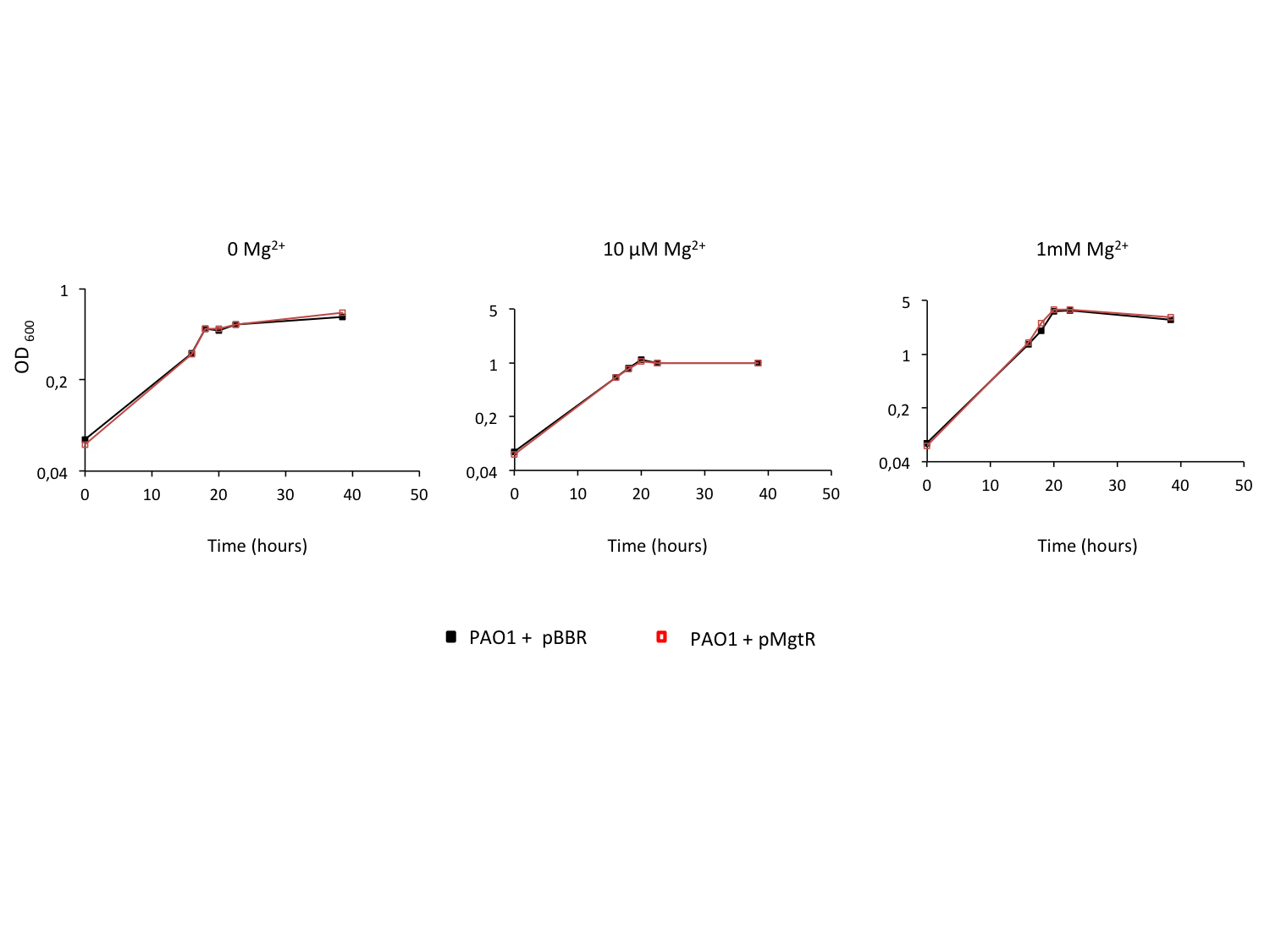

Supplement: S13 Fig — PAO1 strain carrying the pBBR1MCS vector (pBBR) or a pBBR1MCS derivative that encodes mgtR (pMgtR) were grown at 30°C in minimal medium with 0, 10 μM or 1 mM MgSO4. OD600 is indicated over the growth period. The experiment was independently repeated two times and a representative curve is shown. (TIF) [file ppat.1004969.s013.tif]
