# Supplementary material for: A Macrophage Subversion Factor Is Shared by Intracellular and Extracellular Pathogens
Source: PLoS Pathog. 2015 Jun 16;11(6):e1004969. doi: 10.1371/journal.ppat.1004969 (PMC4469704; doi:10.1371/journal.ppat.1004969)
Supplement: S1 Table — (DOC) [file ppat.1004969.s014.doc]

| Strain, plasmid, or oligonucleotide | Genotype, description or sequence | Source and/or reference |
| --- | --- | --- |
| **Strains**  ***E.coli***  Top10 F’  CC118λpir  SM10  ***P. aeruginosa***  PAO1  PAO1Δ*mgtC*  PAO1Δ*mgtC attB::mgtC^+^*  PAO1Δ*pscN*  PAO1Δ*clpV2* | F- *mcrA* Δ*(mrr-hsdRMS-mcrBC)* *φ80lacZΔM15 ΔlacX74 nupG recA1 araD139* Δ(*ara-leu*)7697 *galE15 galK16 rpsL*(Str^R^) *endA1* λ^-^  (λ*pir*) Δ (*ara-leu*), *araD,* Δ*lacX74, galE, galK, phoA-20, thi-1, rpsE, rpoB,* *Arg*(Am), *recA1,* Rfr (λ*pir*)  thi-1, thr, leu, tonA, lacY, supE, recA::RP4-2-Tc::Mu  Wild-type, prototroph, *chl*-2  *mgtC* deletion mutant  complementation of the *mgtC* mutation by a *mgtC* copy inserted at the *att* B site on *P. aeruginosa* chromosome  *pscN* deletion mutant  *clpV2* deletion mutant | Laboratory collection  [1]  Laboratory collection  B. Holloway  This work  This work  [2]  [3] |
| **Plasmids**  pRK2013  pCR2.1  pSBC43  pKNG101  pSBC44  mini-CTX1  pFLP2  pSBC46  pSBC47  pBPF-mCherry  pUT18  pKT25  pKT25-MgtR  pKT25-*St*KdpF  pUT18-*Pa*MgtC  pBBR1MCS  pMgtR | Tra+, Mob+, ColE1, KmR  TA cloning, *lacZ*α, ColE1, f1 ori, ApR KmR  526 bp upstream and 580 bp downstream *mgtC* in pCR2.1  oriR6K, mobRK2, sacBR+, SmR (suicide vector)  *mgtC* deletion of pSBC43 in pKNG101  Ω-FRT-attP-MCS, ori, int, oriT, TcR  Source of Flp recombinase, ApR  525 bp upstream region to the last nucleotide of *mgtC* in pCR2.1  *mgtC* gene of pSBC46 in mini-CTX1  *P. aeruginosa* *oprF* promoter region upstream of the mCherry coding sequence, GmR  AmpR rep_ColE1_  KanR rep_p15A_  Fusion MgtR with T25, KanR rep_p15A_  Fusion *St*KdpF with T25, KanR rep_p15A_  Fusion PaMgtC with T18, AmpR rep_ColE1_  CmR  pBBR1MCS *mgtR* | Laboratory collection  Invitrogen  This work  [4]  This work  [5]  [6]  This work  This work  [7]  [8]  [8]  [9]  [10]  This work  [11]  [9] |
| **Primers**  SBO94  SBO95A  SBO96A  SBO97  SBO98  SBO 99  SBO100  PA4635-18-HindF  PA4635-18-EcoR  PA4635-F  PA4635-R  PA2558-F  PA2558-R  MgtR-F  MgtR-R  16S-F  16S-R  pcrV-F  pcrV-R  gyrA-F  gyrA-R  rpoD-F  rpoD-R | 5’-GCCGGAGAAATGTTCCACT-3’  5’-TCAGTCGGCCGCCAGTTCCATGTTTCACCTCGT-3’  5’-ACGAGGTGAAACATGGAACTGGCGGCCGACTGA-3’  5’-AGCGGTAGCGGACCAGGG-3’  5’-CTGGGCGTACGCGACAGGT-3’  5’-AGCGGGCGCGAGAACTGA-3’  5’-AGCGGTAGCGGACCAGGG-3’  5’-CCCAAGCTTGatggattggaaagtatttc-3’  5’-GGAATTCgtcggccgccagttcgaag-3’  5’-AAAGTATTTCTCCTGCGCGTC-3’  5’-CCGGAGACTACATAGGCGG-3’  5’-GGAGCGCTTCTACCAGATCA-3’  5’-TCTGTTCCACCAGCGAGTC-3’  5’-TGAATCGCTCACCCGATAAAA-3’  5’-GAAAACGATTTGCCAGAGGGC-3’  5’-CAAAACTACTGAGCTAGAGTACG-3’  5’-TAAGATCTCAAGGATCCCAACGGCT-3’  5’-AGTGGGATCTGCGCGAGTT-3’  5’-TGGGTCTGCAGGACATCCTT-3’  5’-AACGACTGGAACAAGCCCTAC-3’  5’-GCGCACGATGGTGTCGTA-3’  5’-GGGCGAAGAAGGAAATGGTC-3’  5’-CAGGTGGCGTAGGTGGAGAA-3’ | This work  This work  This work  This work  This work  This work  This work  This work  This work  This work  This work  This work  This work  This work  This work  [12]  [12]  [13]  [13]  [13]  [13]  [14]  [14] |

1. Herrero M, de Lorenzo V, Timmis KN (1990) Transposon vectors containing non-antibiotic resistance selection markers for cloning and stable chromosomal insertion of foreign genes in gram-negative bacteria. J Bacteriol 172: 6557-6567.

2. Soscia C, Hachani A, Bernadac A, Filloux A, Bleves S (2007) Cross talk between type III secretion and flagellar assembly systems in *Pseudomonas aeruginosa*. J Bacteriol 189: 3124-3132.

3. Sana TG, Hachani A, Bucior I, Soscia C, Garvis S, et al. (2012) The second type VI secretion system of *Pseudomonas aeruginosa* strain PAO1 is regulated by quorum sensing and Fur and modulates internalization in epithelial cells. J Biol Chem 287: 27095-27105.

4. Kaniga K, Delor I, Cornelis GR (1991) A wide-host-range suicide vector for improving reverse genetics in gram-negative bacteria: inactivation of the *blaA* gene of *Yersinia enterocolitica*. Gene 109: 137-141.

5. Hoang TT, Kutchma AJ, Becher A, Schweizer HP (2000) Integration-proficient plasmids for *Pseudomonas aeruginosa*: site-specific integration and use for engineering of reporter and expression strains. Plasmid 43: 59-72.

6. Hoang TT, Karkhoff-Schweizer RR, Kutchma AJ, Schweizer HP (1998) A broad-host-range Flp-FRT recombination system for site-specific excision of chromosomally-located DNA sequences: application for isolation of unmarked *Pseudomonas aeruginosa* mutants. Gene 212: 77-86.

7. Llamas MA, van der Sar A, Chu BC, Sparrius M, Vogel HJ, et al. (2009) A Novel extracytoplasmic function (ECF) sigma factor regulates virulence in *Pseudomonas aeruginosa*. PLoS Pathog 5: e1000572.

8. Karimova G, Pidoux J, Ullmann A, Ladant D (1998) A bacterial two-hybrid system based on a reconstituted signal transduction pathway. Proc Natl Acad Sci U S A 95: 5752-5756.

9. Alix E, Blanc-Potard AB (2008) Peptide-assisted degradation of the *Salmonella* MgtC virulence factor. EMBO J 27: 546-557.

10. Gannoun-Zaki L, Belon C, Dupont C, Hilbert F, Kremer L, et al. (2014) Overexpression of the *Salmonella* KdpF membrane peptide modulates expression of *kdp* genes and intramacrophage growth. FEMS Microbiol Lett 359: 34-41.

11. Kovach ME, Phillips RW, Elzer PH, Roop RM, 2nd, Peterson KM (1994) pBBR1MCS: a broad-host-range cloning vector. Biotechniques 16: 800-802.

12. Matsuda K, Tsuji H, Asahara T, Kado Y, Nomoto K (2007) Sensitive quantitative detection of commensal bacteria by rRNA-targeted reverse transcription-PCR. Appl Environ Microbiol 73: 32-39.

13. Mikkelsen H, Sivaneson M, Filloux A (2011) Key two-component regulatory systems that control biofilm formation in *Pseudomonas aeruginosa*. Environ Microbiol 13: 1666-1681.

14. Savli H, Karadenizli A, Kolayli F, Gundes S, Ozbek U, et al. (2003) Expression stability of six housekeeping genes: A proposal for resistance gene quantification studies of *Pseudomonas aeruginosa* by real-time quantitative RT-PCR. J Med Microbiol 52: 403-408.
