# Supplementary material for: A Macrophage Subversion Factor Is Shared by Intracellular and Extracellular Pathogens
Source: PLoS Pathog. 2015 Jun 16;11(6):e1004969. doi: 10.1371/journal.ppat.1004969 (PMC4469704; doi:10.1371/journal.ppat.1004969)
Supplement: S1 Text — (DOC) [file ppat.1004969.s015.doc]

# Supporting Protocols

**Construction of a *P. aeruginosa mgtC* mutant and complemented strain**

To generate the *mgtC* deletion, 526 bp upstream and 580 bp downstream of the *mgtC* gene were amplified by overlapping PCR with High Fidelity DNA polymerase (ROCHE) using SBO94, SBO95A, SBO96A, and SBO97 primers (S1 Table) [1]. The PCR product was cloned in pCR2.1 (TA-cloning KIT, Invitrogen) giving pSBC43, which was then sequenced (GATC) and subcloned in pKNG101 suicide vector giving the mutator pSBC44. pSBC44, maintained in the *E. coli* CC118λpir strain, was mobilized in the wild-type *P. aeruginosa* strain PAO1. The mutants, with a double recombination event resulting in the non-polar deletion of the *mgtC* gene, were verified by PCR using external primers SBO98-SBO100.

Complementation by a single copy of the *mgtC*^+^ gene under its own promoter was carried out with the miniCTX integration method at *attB* region [2]. The entire *mgtC* gene with the 525 bp-upstream region in which a σ^70^ dependent promoter was predicted (S1 Fig) was amplified with SBO94 and SBO99, cloned in the pCR2.1 giving pSBC46, which was then sequenced and subcloned in mini-CTX1 plasmid vector giving pSBC47. Chromosomal insertion of the mini-CTX construct pSBC47 carrying *mgtC*^+^ was obtained via a mating between the recipient PAO1Δ*mgtC* (grown O/N at 42 °C), and *E. coli* SM10 pSBC47, with selection on PIA for tetracycline. Finally, excision of the unwanted plasmid DNA sequences was achieved by producing the Flp recombinase from a conjugative plasmid, pFLP2. This step results in an unmarked integrant. The pFLP2 plasmid was cured on sucrose 6%.

# Swimming and twitching motility assays

For motility assays, O/N cultures were done in MM63 medium supplemented with glucose, Casamino acids and 1mM MgSO_4_. For twitching motility, plates containing 1.5% agar were inoculated by stabbing the agar plates. After 24 h growth at 30 °C, the agar was removed and the plates were stained with 1% crystal violet for 10 min and washed with water. Spreading of bacteria from the inoculation point could be observed by the size of the stained surface on the plastic. For swimming assays, 0.3% agar plates were inoculated and were incubated 5 h at 37°C. The spreading of the colony on the agar could readily be observed.

**Zebrafish maintenance**

Zebrafish experiments were performed using the *golden* zebrafish mutant [3] purchased from a local company (Antinea SARL, Montpellier, France) and maintained according to standard described procedures [4]. The Tg(*mpeg1*::*mCherry*) zebrafish line, which was generated as described below, was used for antisense experiments. Eggs were obtained by natural spawning and incubated at 28.5 °C in “fish water” (water with NaCl 60 μg/mL). Ages of the embryos are expressed as hours post fertilization (hpf).

**Generation of transgenic (*mpeg1*::*mCherry*) line**

The Mpeg1 promoter is used to drive the specific expression of membrane targetted redfluorescent protein in macrophages. Amplificating primers for fragment of Mpeg1 promoter were zMpeg1P4 (TTGGAGCACATCTGAC) and zMpeg1E2N2 (TTATAGCGGCCGCGAAATGCTCTTGACTTCATGA), digested by NotI and ligated to the coding phase of the farnesylated mCherry protein so that the Mpeg1 AUG would be in phase with the downstream mCherry-F open reading frame on a Tol2 derived vector. The resulting plasmid was injected together with the Transposase mRNA in one cell stage embryos.

**Microinjection of Morpholinos into embryos**

A mix of splice-blocking (GGTCTTTCTCCTTACCATGCTCTCC) and ATG (CCTCCATTCTGTACGGATGCAGCAT) morpholinos (MO) against *pu.1* gene were purchased from Gene Tools and combined as described [5] [6]. MO mix were prepared in MO buffer containing 10 % phenol red and injected (3 nl) into one cell stage tg(*mpeg1*::*mCherry*) using a Tritech Research digital microINJECTOR (MINJ-D). The correct depletion of macrophages cells in *pu.1* morphants was confirmed by fluorescent microscopy observation.

**Fluorescence Microscopy**

Fluorescence live microscopy of embryos are using an Olympus MVX10 epifluorescent microscope equipped with a X-Cite®120Q (Lumen Dynamics) 120W mercury light source. Images are acquired with a digital color camera (Olympus XC50) and processed using CellSens (Olympus). Fluorescence filters cubes TRITC-MVX10 are used for detection of red light. Final images analysis and visualization are performed using GIMP 2.6 freeware to adjust levels and brightness and to remove out-of-focus background fluorescence.

1. Vasseur P, Vallet-Gely I, Soscia C, Genin S, Filloux A (2005) The pel genes of the *Pseudomonas aeruginosa* PAK strain are involved at early and late stages of biofilm formation. Microbiology 151: 985-997.

2. Hoang TT, Kutchma AJ, Becher A, Schweizer HP (2000) Integration-proficient plasmids for *Pseudomonas aeruginosa*: site-specific integration and use for engineering of reporter and expression strains. Plasmid 43: 59-72.

3. Lamason RL, Mohideen MA, Mest JR, Wong AC, Norton HL, et al. (2005) SLC24A5, a putative cation exchanger, affects pigmentation in zebrafish and humans. Science 310: 1782-1786.

4. Westerfield M (2007) In: The zebrafish book: a guide for the laboratory use of the zebrafish (*Danio rerio).* 5th edition, Eugene, University of Oregon Press. Paperback.

5. Clay H, Davis JM, Beery D, Huttenlocher A, Lyons SE, et al. (2007) Dichotomous role of the macrophage in early *Mycobacterium marinum* infection of the zebrafish. Cell Host Microbe 2: 29-39.

6. Bernut A, Herrmann JL, Kissa K, Dubremetz JF, Gaillard JL, et al. (2014) *Mycobacterium abscessus* cording prevents phagocytosis and promotes abscess formation. Proc Natl Acad Sci U S A 111: E943-952.
